# Transcriptomic and Metabolomic Analyses Reveal Intestinal Lipid Metabolic Disturbance in Loaches Co-Exposed to Polystyrene Nanoplastics and Imidacloprid

**DOI:** 10.3390/biology15181608

**Published:** 2026-09-11

**Authors:** Yingbing Su, Shenghao Wang, Jiali Liu, Haishan Cheng, Hongtao Liao, Ziyan Yang, Yumeng Ban, Pupu Yan, Liwei Guo, Daiqin Yang

**Affiliations:** 1 College of Animal Science and Technology, Yangtze University, Jingzhou 434025, China; su-yingbing@163.com (Y.S.); 2025710981@yangtzeu.edu.cn (S.W.); 2023721005@yangtzeu.edu.cn (H.C.);; 2 Fujian Key Laboratory of Veterinary Chinese Medicine and Animal Health, College of Animal Science, Fujian Agriculture and Forestry University, Fuzhou 350002, China; liujiali@fafu.edu.cn; 3 National Reference Laboratory for Veterinary Drug Residue Testing, Huazhong Agricultural University, Wuhan 430070, China

**Keywords:** polystyrene nanoplastics, imidacloprid, combined exposure, *Misgurnus anguillicaudatus*, oxidative stress, transcriptomics, metabolomics, lipid metabolism

## Abstract

Polystyrene nanoplastics (PS-NPs; nominal diameter, 100 nm; unmodified and negatively charged) and imidacloprid (IMI) are common freshwater contaminants, but little is known about their combined toxicity in benthic fish. In this study, loaches (*Misgurnus anguillicaudatus*) were exposed for 21 days to clean water, 100 μg/L PS-NPs, 78 μg/L IMI, or 78 μg/L IMI combined with 25, 50, or 100 μg/L PS-NPs. Each treatment contained 90 fish distributed among three replicate tanks. Survival, hepatic oxidative-stress indices, inflammatory gene expression, histopathology, and intestinal transcriptomic and metabolomic profiles were evaluated. Survival decreased across the co-exposure groups, with a significant overall difference among treatments (log-rank test, *p* = 0.0027). Hepatic CAT, SOD, and GSH levels decreased, whereas MDA increased, with the strongest changes observed in the higher PS-NP co-exposure groups. Pro-inflammatory genes, including *IL-1β*, *IL-6*, *IL-8*, and *TNF-α*, were upregulated, whereas *IL-4* and *IL-10* were downregulated. Histopathological injury of the gill, intestine, and liver was most pronounced in the NIH group. Transcriptomic analysis identified changes in pathways related to digestion, cell-cycle regulation, cholesterol metabolism, and lipid metabolism, while metabolomic analysis revealed alterations in glycerophospholipid, sphingolipid, fatty-acid, and carbohydrate metabolism. *HMGCS1*, *CEL*, *CYP8B1*, *APOB*, and *APOC1* showed treatment-associated expression changes, with APOB displaying a non-linear response across the co-exposure groups. Overall, PS-NP co-exposure was associated with stronger IMI-related adverse responses under the tested conditions and substantial disruption of intestinal lipid homeostasis.

## Simple Summary

Polystyrene nanoplastics and the insecticide imidacloprid can occur together in freshwater. We examined the biological effects of their co-exposure in loaches. Fish were exposed for 21 days to either contaminant alone or to imidacloprid combined with three concentrations of polystyrene nanoplastics. Co-exposure was associated with lower survival, reduced antioxidant capacity, enhanced inflammatory responses, and greater injury in the gills, intestine, and liver. Intestinal transcriptomic and metabolomic analyses further revealed disturbances in digestive and lipid-metabolic processes. Quantitative PCR confirmed treatment-associated changes in five lipid-related genes. These findings indicate that the presence of polystyrene nanoplastics may enhance IMI-related adverse effects under the tested conditions and that disruption of intestinal lipid homeostasis is an important component of the co-exposure response.

## 1. Introduction

Plastic pollution and pesticide runoff are major and increasingly interconnected pressures on freshwater ecosystems [1,2]. Global plastic emissions are predicted to rise substantially without stronger mitigation, while neonicotinoid insecticides are frequently detected in surface waters near agricultural areas [3]. Freshwater biodiversity is therefore exposed not only to individual contaminants but also to complex mixtures whose biological effects may differ from those predicted from single-compound tests [4]. Because the gastrointestinal tract and gills directly contact contaminated water and suspended particles, fish are particularly vulnerable to particle-associated toxicants [5]. A recent synthesis showed that micro- and nanoplastics can impair fish intestinal morphology, barrier function, oxidative balance, and microbial homeostasis [6].

Polystyrene nanoplastics are widely used as model particles for investigating the biological effects of nanoscale plastic contamination [7]. Their small size facilitates ingestion, tissue distribution, and interaction with cellular membranes [8]. Experimental exposure has produced tissue accumulation and biochemical toxicity in freshwater fish [9], hepatic lipid metabolic disruption in large yellow croaker [10], and disturbances of the brain–intestine–microbiota axis in zebrafish [11]. Omics-based studies further indicate that nanoplastic exposure can alter hepatic energy and lipid metabolism [12]. Imidacloprid, one of the most extensively used neonicotinoid insecticides, is persistent enough to reach non-target aquatic organisms. Its toxicity has been linked to oxidative stress and metabolic disruption [13], developmental toxicity in fish [14], oxidative DNA damage [15], and intestinal injury mediated by inflammatory signaling and impaired junctional integrity [16].

Nanoplastics may modify the fate and bioavailability of coexisting organic pollutants through surface adsorption, transport, and desorption. Polystyrene nanoplastics showed strong sorption toward hydrophobic organic pollutants [17]. In addition, nanoscale polystyrene particles have been shown to facilitate the transport and uptake of phenanthrene in zebrafish embryos compared with larger plastic particles [18]. These findings suggest that particle size and sorption behavior may modify the bioavailability of coexisting organic pollutants. Co-exposure to plastic particles and imidacloprid has aggravated hepatic and intestinal effects in mice and increased toxicity in earthworms [19,20]. Adsorption studies also support interactions between neonicotinoids and plastic-derived particles [21]. In fish, combined exposure to microplastics and another organic pollutant caused intestinal damage, oxidative stress, and microbial dysbiosis [22]. Nevertheless, evidence remains limited for benthic fish that live at the sediment–water interface and are chronically exposed through both water and feeding.

The loach (*Misgurnus anguillicaudatus*) is a benthic freshwater fish that is readily exposed to contaminants in water and sediment. In the present study, we investigated whether co-exposure to PS-NPs was associated with stronger IMI-related adverse responses in loaches. The exposure concentrations used in the present study were selected as experimental sublethal concentrations rather than as direct representations of typical environmental concentrations. The IMI concentration of 78 μg/L was selected with reference to our previous study in loach, in which a similar concentration was used to characterize sublethal intestinal toxicity. PS-NP concentrations of 25, 50, and 100 μg/L were used to establish a graded co-exposure series and to examine whether increasing PS-NP levels were associated with stronger IMI-related responses under controlled experimental conditions. Survival, oxidative-stress indices, inflammatory gene expression, and tissue histology were examined under single- and co-exposure conditions. Intestinal transcriptomics and metabolomics were then used to identify the main biological processes associated with the observed injury, and selected lipid-related genes were validated by quantitative polymerase chain reaction.

## 2. Materials and Methods

### 2.1. Experimental Animals, Chemicals, and Husbandry

Healthy loaches (*Misgurnus anguillicaudatus*; body mass 26–32 g; body length 13–18 cm) were obtained from the Taihu Aquaculture Base (Jingzhou, China). Before the experiment, fish were acclimated for 7 days in aerated laboratory water at 22–27 °C and were fed a commercial diet twice daily. Imidacloprid (IMI; CAS No. 138261-41-3; purity ≥99%; Tianjin Siansi Biochemical Technology Co., Ltd., Tianjin, China) was stored at 4 °C. Monodisperse, unmodified polystyrene nanoplastics (PS-NPs; nominal diameter, 100 nm; negative surface charge; 25 mg/mL aqueous suspension) were obtained from Tianjin Base-line ChromTech Research Centre (Tianjin, China). Before use, the PS-NP stock suspension was sonicated at 40 kHz for 10 min and diluted with deionized water. Exposure solutions were freshly prepared, and 80% of the water was renewed every 2 days. Fish were fed twice daily, and mortality was recorded each day. The animal study protocol was approved by the Animal Ethics Committee of Yangtze University (approval code: YZ20250622-FAG; approval date: 22 June 2025).

### 2.2. Exposure Design and Sample Collection

**Figure 1:**
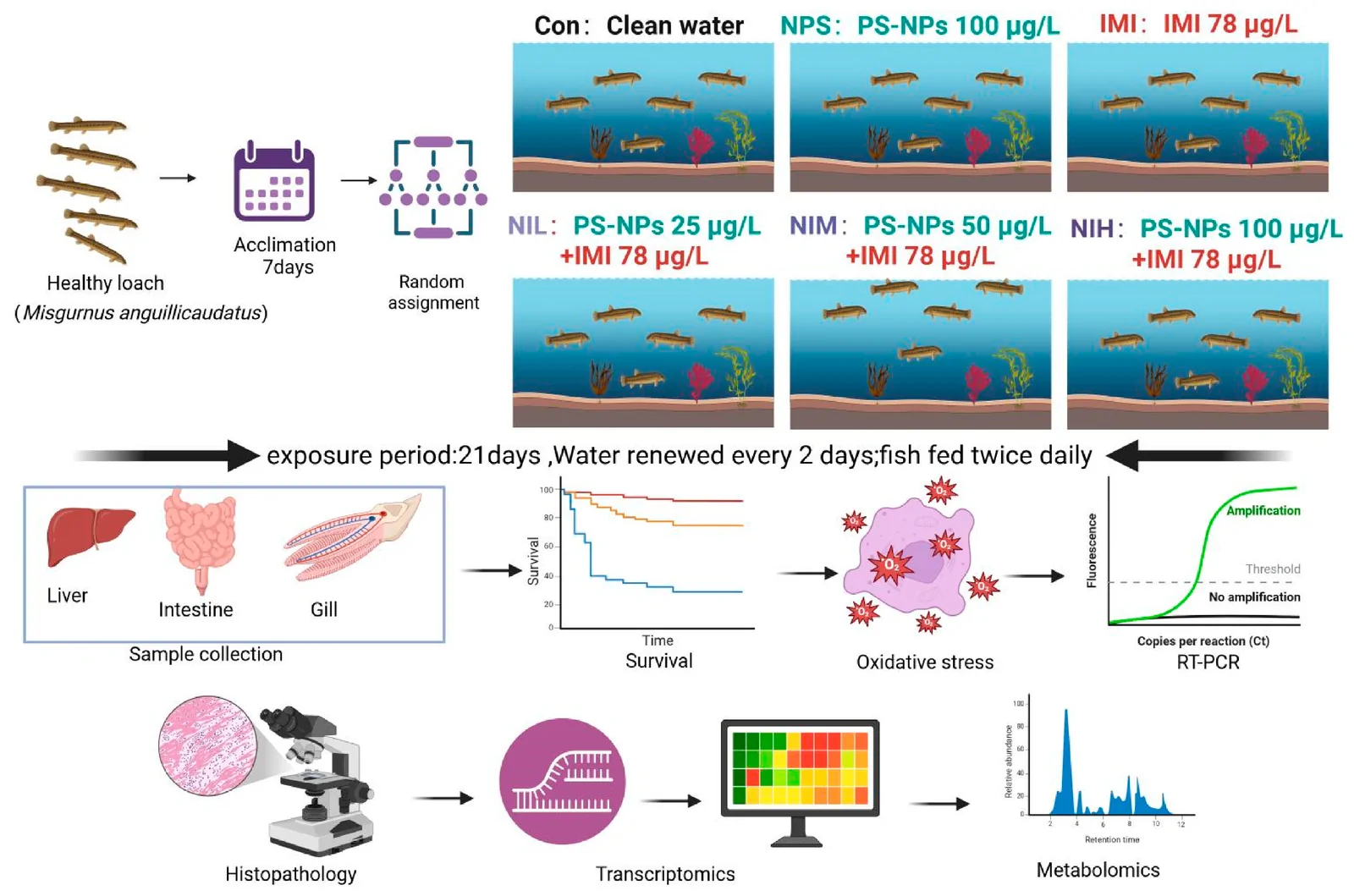
Experimental design. Healthy loaches were acclimated for 7 days and then assigned to six groups for a 21-day waterborne exposure. Each treatment contained 90 fish in three replicate tanks (30 fish per tank). Water was renewed every 2 days and fish were fed twice daily. Survival was monitored throughout the 21-day exposure. Liver, intestinal, and gill tissues were collected for oxidative-stress assays, gene-expression analysis, histopathology, intestinal transcriptomics, and intestinal metabolomics. CON, control; NPS, 100 μg/L polystyrene nanoplastics; IMI, 78 μg/L imidacloprid; NIL, NIM, and NIH, 78 μg/L imidacloprid combined with 25, 50, and 100 μg/L polystyrene nanoplastics, respectively. The schematic was created with BioRender.com (https://www.biorender.com/).

The IMI concentration of 78 μg/L was selected with reference to our previous exposure study in loach, in which 73 μg/L IMI was evaluated as a sublethal exposure concentration [23]. After acclimation, 540 loaches were randomly assigned to six treatments. Each treatment contained 90 fish distributed among three replicate tanks (30 fish per tank). Fish were exposed for 21 days to clean water (CON), 100 μg/L PS-NPs (NPS), 78 μg/L IMI (IMI), 78 μg/L IMI plus 25 μg/L PS-NPs (NIL), 78 μg/L IMI plus 50 μg/L PS-NPs (NIM), or 78 μg/L IMI plus 100 μg/L PS-NPs (NIH) (Figure 1 and Table 1). The positions of the tanks were randomized. At the end of the exposure, fish were anesthetized with 100 mg/L MS-222 (Merck KGaA, Darmstadt, Germany). Liver, intestine, and gill tissues were collected on ice. Samples for histology were fixed in 4% paraformaldehyde. Samples used for biochemical, gene-expression, transcriptomic, and metabolomic analyses were immediately frozen in liquid nitrogen and stored at −80 °C. Samples for each endpoint were collected across the three replicate tanks; for RNA sequencing and metabolomics, one biological sample from each tank was analyzed (*n* = 3 per treatment). All exposure concentrations reported in this study were nominal concentrations. The hydrodynamic size distribution, zeta potential, particle-number concentration, and aggregation state of PS-NPs in the exposure water were not monitored during the 21-day exposure. In addition, dissolved and particle-associated IMI concentrations were not analytically quantified.

### 2.3. Hepatic Oxidative-Stress Biomarkers

Approximately 0.1 g of liver tissue was homogenized in the extraction buffer supplied with the corresponding assay kit. Homogenates were centrifuged at 8000× *g* for 10 min at 4 °C, and the supernatants were analyzed for catalase (CAT; AKAO003-1M), superoxide dismutase (SOD; AKAO001M-200S), reduced glutathione (GSH; AKPR008M), and malondialdehyde (MDA; AKFA013M) using commercial kits (Beijing Boxbio Science & Technology Co., Ltd., Beijing, China) according to the manufacturer’s instructions. All results were normalized to the wet mass of liver tissue. CAT activity was calculated from the decrease in absorbance associated with H_2_O_2_ decomposition and expressed as U/g tissue. SOD activity was determined using the xanthine oxidase–nitroblue tetrazolium inhibition method and expressed as U/g tissue. GSH content was quantified from a GSH standard curve and expressed as μg/g tissue. MDA content was determined using the thiobarbituric acid method and expressed as nmol/g tissue. All calculations were performed according to the equations supplied by the manufacturer.

### 2.4. RNA Extraction and Quantitative Real-Time PCR

Total RNA was isolated from liver or intestinal tissue using TRIzol reagent (BS258A; Biosharp, Hefei, China). RNA concentration and purity were assessed with a NanoDrop 2000 spectrophotometer (Thermo Fisher Scientific, Waltham, MA, USA). Complementary DNA was synthesized using a reverse-transcription kit (ABclonal Technology, Wuhan, China), and quantitative real-time PCR was performed with SYBR Green chemistry on a CFX96 system (Bio-Rad Laboratories, Hercules, CA, USA). The cycling program comprised initial denaturation followed by 30 cycles of 95 °C for 5 s and 60 °C for 30 s. For each treatment, qPCR analysis included six biological samples obtained from six individual fish, with two fish sampled from each of the three replicate tanks (*n* = 6 per treatment). Each biological sample was analyzed in three technical replicate reactions. Relative transcript abundance was calculated using the 2^−ΔΔCt^ method, with GAPDH as the internal reference gene and CON as the calibrator group. All primers were synthesized by Sangon Biotech (Shanghai, China) (primer sequences are listed in Table 2). The stability of GAPDH expression across treatments was evaluated by comparing its raw Ct values among groups using one-way ANOVA and by calculating the coefficient of variation across all biological samples. No significant treatment-associated difference in GAPDH Ct values was detected (*p* = 0.452, coefficient of variation of 2.1%), and the low coefficient of variation supported its use as the reference gene. Amplification specificity was confirmed by the presence of a single melt-curve peak for each primer pair, and no amplification was observed in the no-template controls. Hepatic *IL-1β*, *IL-6*, *IL-8*, *TNF-α*, *IL-4*, and *IL-10* were quantified to characterize inflammatory responses, whereas intestinal *HMGCS1*, *CEL*, *CYP8B1*, *APOB*, and *APOC1* were quantified to validate the transcriptomic results. Amplification efficiency was evaluated using serial dilutions of pooled cDNA. Standard curves were generated by linear regression of Ct values against the logarithm of relative template concentration, and amplification efficiency was calculated as E (%) = [10 ^(−1/slo^pe) − 1] × 100. Amplification efficiencies ranged from 94.99% to 101.48%, with R^2^ values ranging from 0.9763 to 0.9992 (Supplementary Table S2).

### 2.5. Histopathological Examination

Fixed gill, intestinal, and liver samples were dehydrated through graded ethanol, cleared in xylene, embedded in paraffin, sectioned, and stained with hematoxylin and eosin. Sections were examined using a light microscope (XD30A-RFL; Sunny Optical Technology, Yuyao, China). Representative fields were recorded at 4× and 20× objective magnification. Histological interpretation focused on gill filament architecture, epithelial proliferation and fusion, inflammatory infiltration, tissue vacuolation, intestinal mucosal integrity, hepatocyte organization, and necrotic change. Histopathological lesions were assessed semi-quantitatively in four fish per treatment. For each fish, three to five non-overlapping 20× fields were evaluated for each tissue. Lesion severity was scored using a four-point scale: 0, no obvious lesion; 1, mild focal change; 2, moderate lesion; and 3, severe or extensive architectural disruption or inflammatory change. Field-level scores were averaged to obtain one fish-level score for each tissue. Fish-level scores (*n* = 4 per treatment) were summarized descriptively as mean ± SD, median, and range. Individual microscopic fields were not treated as independent biological replicates.

### 2.6. Intestinal Transcriptome Sequencing and Bioinformatic Analysis

Intestinal tissue from one fish in each replicate tank was used for RNA sequencing (n = 3 per treatment). After RNA quality assessment with the NanoDrop 2000 and Agilent 2100 Bioanalyzer (Agilent Technologies, Santa Clara, CA, USA), polyadenylated RNA was enriched using oligo(dT) magnetic beads and fragmented to approximately 300 bp. First- and second-strand complementary DNA synthesis, PCR enrichment, size selection, and library quality control were then performed. Indexed libraries were pooled, denatured, and subjected to paired-end 150-bp sequencing on an Illumina HiSeq platform.

Raw reads were processed with Cutadapt v2.6 to remove adapter sequences and reads with mean quality scores below Q20. Across the 18 libraries, an average of 47.35 million clean reads per sample was obtained, with a mean mapping rate of 89.45% (range, 80.70–90.79%). Clean reads were aligned using a Bowtie2 index followed by TopHat2 alignment, allowing a maximum of two mismatches, to the NCBI RefSeq assembly HAU_Mang_1.0 (accession GCF_027580225.1). The corresponding NCBI RefSeq annotation release GCF_027580225.1-RS_2023_04 was used for gene-level quantification. Genes with zero total counts across the six samples included in a given exposure-versus-control contrast were excluded before differential-expression analysis. Gene-level counts were analyzed using DESeq2 v1.22.2. No batch covariate was included because no batch variable was recorded in the supplied sequencing metadata. Differentially expressed genes were defined using |log2(fold change)| > 1 and Benjamini–Hochberg-adjusted *p* < 0.05. Gene Ontology enrichment was performed using topGO, and Kyoto Encyclopedia of Genes and Genomes pathway analysis was performed using KOBAS v3.0. *p* values were adjusted across all tested terms or pathways within each comparison using the Benjamini–Hochberg procedure, and adjusted *p* < 0.05 was used to define significant enrichment.

### 2.7. Intestinal Untargeted Metabolomics

Intestinal tissue (25 mg) was extracted with 500 μL methanol/acetonitrile/water (2:2:1, *v*/*v*/*v*) containing isotope-labeled internal standards. Samples were vortexed for 30 s, homogenized at 35 Hz for 4 min, sonicated in an ice-water bath for 5 min, and subjected to three extraction cycles. After incubation at −40 °C for 1 h, extracts were centrifuged at 12,000× *g* for 15 min at 4 °C. Equal aliquots of all supernatants were combined to prepare quality-control samples.

Chromatographic separation was performed using a Vanquish ultra-high-performance liquid chromatograph coupled to an Orbitrap Exploris 120 mass spectrometer (Thermo Fisher Scientific). Polar metabolites were separated on an ACQUITY UPLC BEH Amide column (2.1 × 50 mm, 1.7 μm; Waters, Milford, MA, USA). Mobile phase A consisted of water containing 25 mmol/L ammonium acetate and 25 mmol/L ammonia; mobile phase B was acetonitrile. The autosampler was maintained at 4 °C and the injection volume was 2 μL. Electrospray ionization was performed in positive and negative modes using the following settings: sheath gas, 50 arbitrary units; auxiliary gas, 15 arbitrary units; capillary temperature, 320 °C; full-scan resolution, 60,000; MS/MS resolution, 15,000; stepped normalized collision energies, 20/30/40; spray voltage, 3.8 kV in positive mode and −3.4 kV in negative mode.

Raw files were converted to mzXML format using ProteoWizard v3.0. Peak detection, alignment, normalization, and annotation were performed using an in-house R workflow and BiotreeDB v3.0. For each exposure-versus-control comparison, raw *p* values were calculated using a two-sided Welch’s t-test on log2-transformed normalized feature intensities. Pairwise orthogonal partial least-squares discriminant analysis (OPLS-DA) was performed using SIMCA v16.0.2 (Sartorius Stedim Data Analytics AB, Umeå, Sweden). Model performance was evaluated using R^2^Y and Q^2^ obtained by leave-one-out cross-validation, and potential model overfitting was assessed using 200 permutation tests. Variable importance in projection (VIP) scores were extracted from the corresponding validated OPLS-DA models. Raw *p* < 0.05 was considered nominally significant. To control for multiple testing, raw *p* values were adjusted across all 58,279 tested LC–MS feature records within each comparison using the Benjamini–Hochberg procedure. Differential feature records were ultimately defined by VIP > 1 and Benjamini–Hochberg q < 0.05. In the volcano plots, the *x*-axis represents log2(fold change), and the *y*-axis represents −log10(Benjamini–Hochberg q). Metabolite-annotation confidence was classified according to the Metabolomics Standards Initiative criteria. Features supported by MS/MS library matches in BiotreeDB v3.0 but not confirmed using authentic standards were classified as Level 2 putative annotations. Class-level assignments were classified as Level 3, whereas features lacking sufficient structural evidence were retained as Level 4 unknowns. No metabolite was assigned to Level 1 because authentic-standard and retention-time confirmation under identical analytical conditions was unavailable. Annotated features were subsequently mapped to Kyoto Encyclopedia of Genes and Genomes pathways.

### 2.8. Integrated Transcriptome–Metabolome Analysis

For each exposure-versus-control comparison, Pearson correlation coefficients were calculated between significantly altered intestinal transcripts and FDR-selected metabolic features. Correlation matrices were visualized as clustered heatmaps. Genes consistently connected with lipid-, bile-acid-, digestive-, and immune-related metabolites were prioritized, and a protein–protein interaction network was constructed to identify highly connected candidates for quantitative PCR validation.

### 2.9. Statistical Analysis

Data are presented as mean ± standard deviation (SD). For the hepatic oxidative-stress endpoints, the replicate tank was treated as the experimental unit, and measurements obtained from fish sampled within the same tank were averaged before statistical analysis. Normality of residuals was evaluated using the Shapiro–Wilk test, and homogeneity of variance was assessed using Levene’s test. Endpoints satisfying the assumptions of parametric analysis were analyzed using one-way ANOVA followed by Tukey–Kramer multiple comparisons; where the assumption of equal variance was not satisfied, Welch’s ANOVA with Games–Howell multiple comparisons was used. Survival analysis included all 90 fish per treatment. Survival curves were estimated using the Kaplan–Meier method and compared among treatments using the log-rank (Mantel–Cox) test. Statistical significance was defined as *p* < 0.05. Statistical analyses were performed using IBM SPSS Statistics v27.0 and GraphPad Prism v9.5. Statistical procedures for the transcriptomic and metabolomic datasets are described in the corresponding sections.

## 3. Results

### 3.1. Effects of Co-Exposure on Survival, Oxidative Stress, and Inflammation

**Figure 2:**
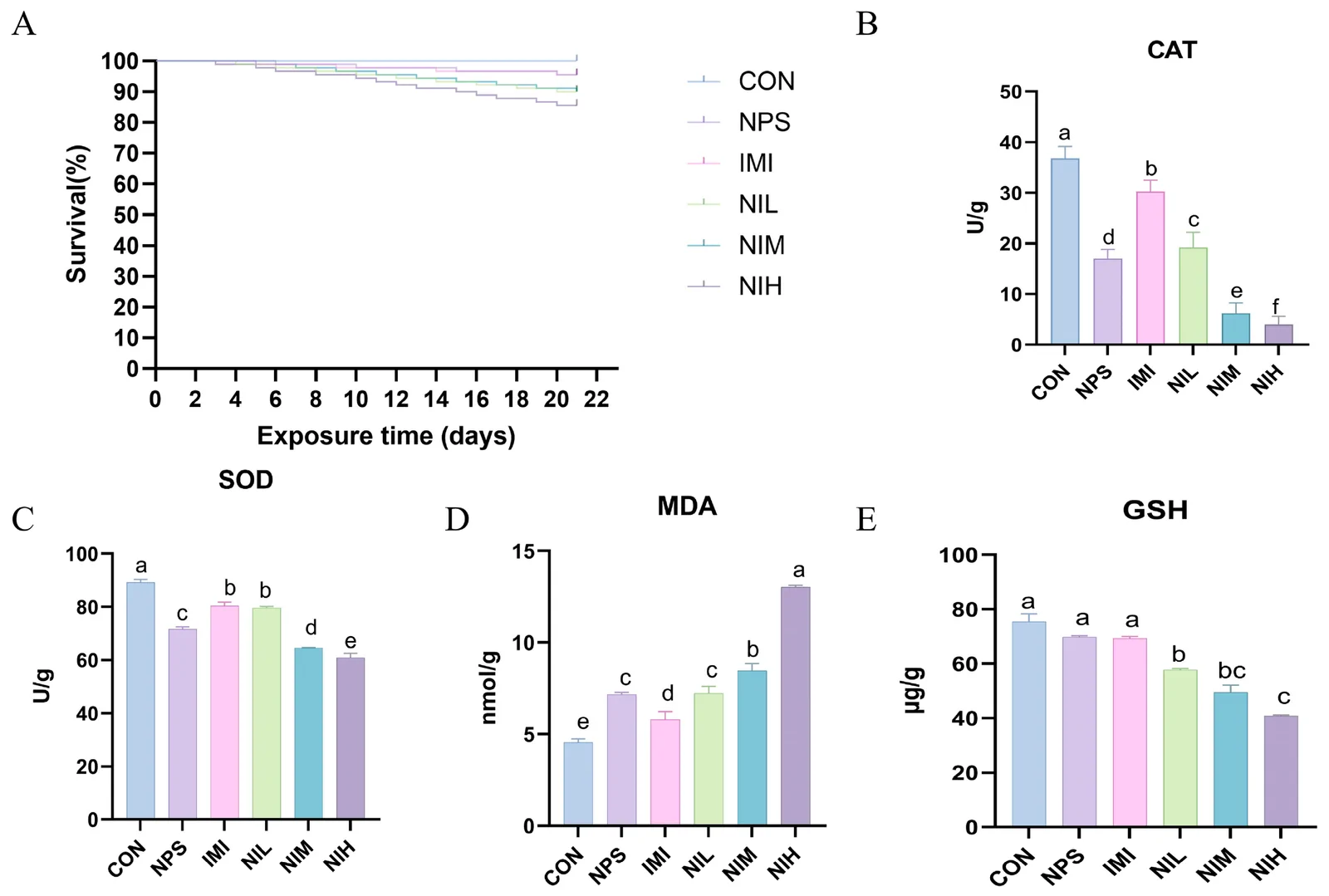
Survival and hepatic oxidative-stress responses of loaches after 21 days of exposure. (**A**) Kaplan–Meier survival curves based on 90 fish per treatment. Differences among groups were evaluated using the log-rank (Mantel–Cox) test (χ^2^ = 18.19, df = 5, *p* = 0.0027). (**B**) Catalase (CAT) activity. (**C**) Superoxide dismutase (SOD) activity. (**D**) Malondialdehyde (MDA) content. (**E**) Reduced glutathione (GSH) content. For biochemical endpoints, measurements from fish sampled within the same tank were averaged to obtain tank-level values, and the replicate tank was treated as the experimental unit. Data are presented as mean ± SD. Different lowercase letters indicate significant differences among treatments. CAT, SOD, and MDA were analyzed using one-way ANOVA followed by Tukey–Kramer multiple comparisons, whereas GSH was analyzed using Welch’s ANOVA followed by Games–Howell multiple comparisons (*p* < 0.05).

No mortality occurred in the CON group during the 21-day exposure. Final survival was 95.2% in both the NPS and IMI groups, 90.5% in the NIL and NIM groups, and 85.7% in the NIH group. Survival decreased across the co-exposure groups, and the overall difference among treatments was statistically significant (log-rank test, χ^2^ = 18.19, df = 5, *p* = 0.0027; Figure 2A).

Hepatic oxidative-stress biomarkers differed significantly among treatments (Figure 2B–E). CAT, SOD, and GSH were generally lower in the exposure groups than in CON, whereas MDA was increased. The strongest changes were observed in the higher PS-NP co-exposure groups. NIH showed the lowest CAT, SOD, and GSH values and the highest MDA level, indicating a pronounced disturbance of hepatic oxidative balance under the tested co-exposure conditions. Detailed overall test statistics, pairwise mean differences, adjusted *p* values, and 95% confidence intervals are provided in Supplementary Table S1.

**Figure 3:**
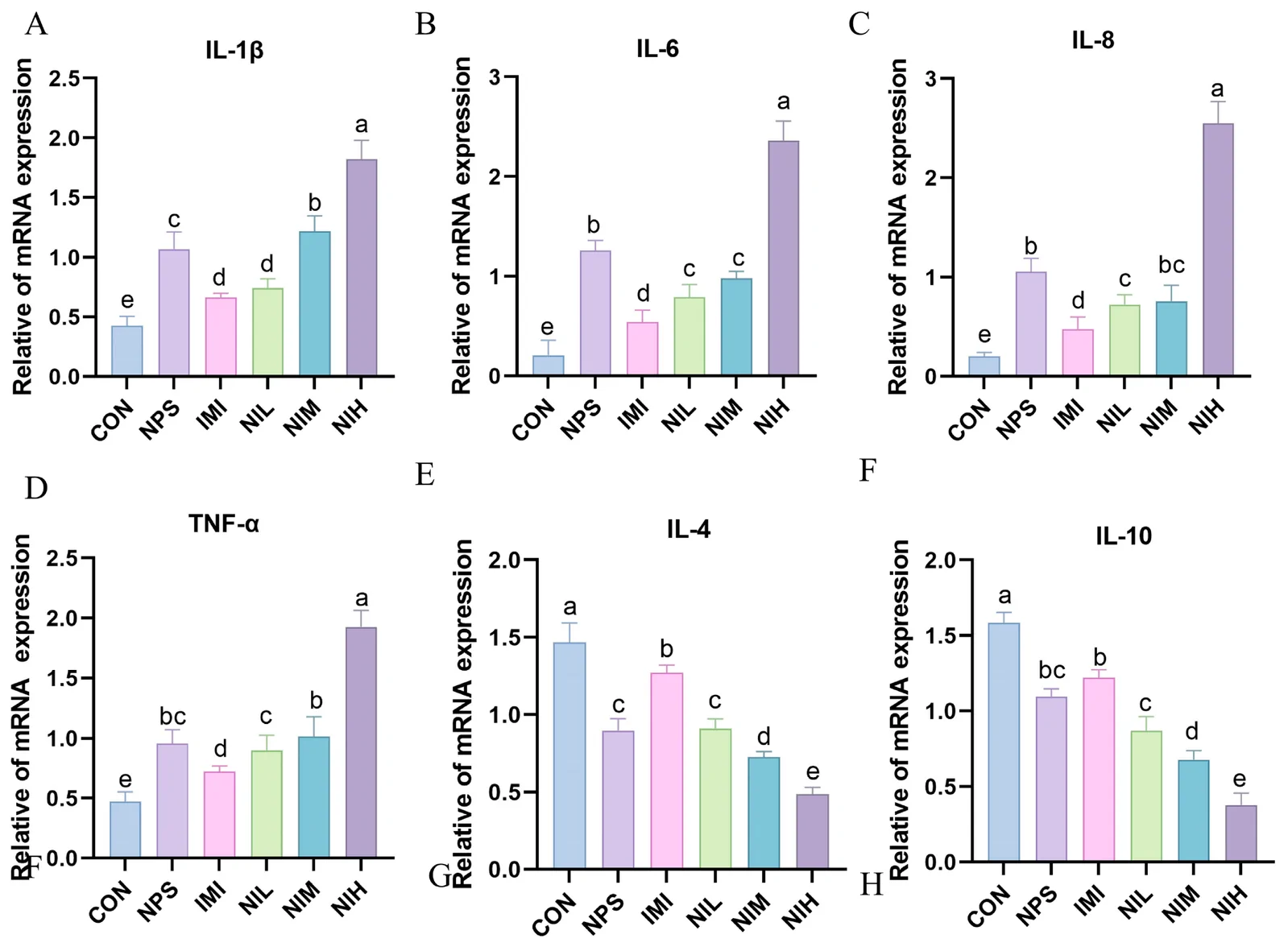
Hepatic inflammatory gene expression after 21 days of exposure. Relative mRNA expression of (**A**) *IL-1β*, (**B**) *IL-6*, (**C**) *IL-8*, (**D**) *TNF-α*, (**E**) *IL-4*, and (**F**) *IL-10*. Data are presented as mean ± standard deviation. Bars with different lowercase letters indicate significant differences among groups (*p* < 0.05).

Consistent with these oxidative-stress responses, Figure 3 show the relative mRNA expression of six cytokines across the same seven groups (CON, NPS, IMI, NIL, NIM, NIH). Pro-inflammatory cytokines (*IL-1β*, *IL-6*, *IL-8*, *TNF-α*) were lowest in CON and highest in NIH, while anti-inflammatory cytokines (*IL-4*, *IL-10*) showed the opposite trend, being highest in CON and lowest in NIH. Different letters above the bars indicate significant differences among groups (*p* < 0.05).

### 3.2. Histological Changes in the Gill, Intestine, and Liver

**Figure 4:**
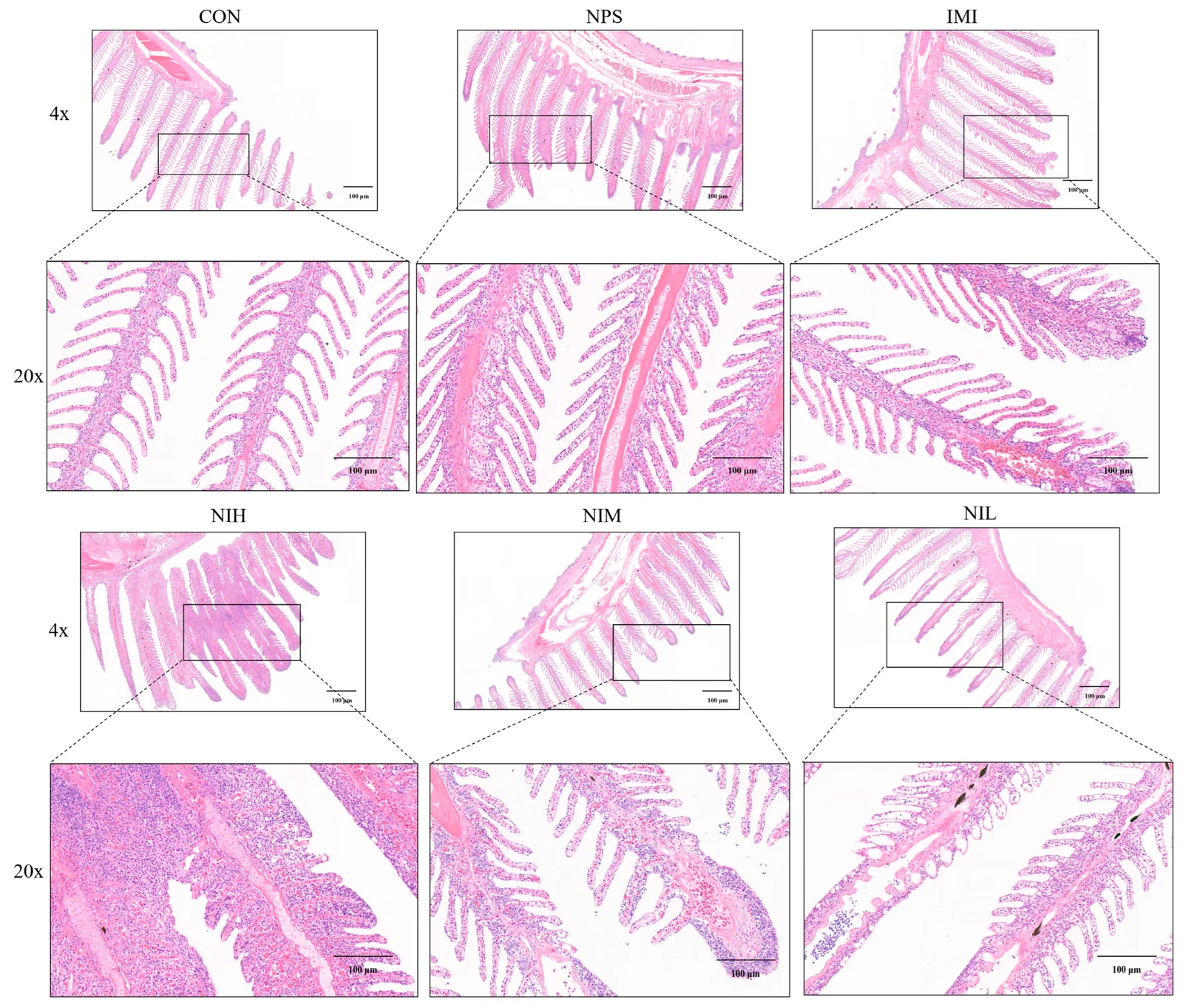
Representative hematoxylin-and-eosin-stained gill sections after 21 days of exposure. Low- and high-magnification views were obtained using 4× and 20× objectives, respectively. Boxes in the low-magnification images indicate the regions shown below. Co-exposure caused progressively greater filament curvature, basal proliferation and fusion, vacuolation, and inflammatory infiltration, with the most extensive injury in NIH. Scale bars = 100 μm.

Gill filaments in the CON group were regularly arranged, with intact secondary lamellae and no evident inflammatory infiltration or vacuolation (Figure 4). NPS and IMI exposure caused curvature, basal proliferation, focal fusion, and vacuolar change. Lesions became more extensive in the co-exposure groups, including pronounced filament distortion, epithelial proliferation, fusion, inflammatory infiltration, and loss of normal lamellar architecture. NIH displayed the most severe structural disruption.

**Figure 5:**
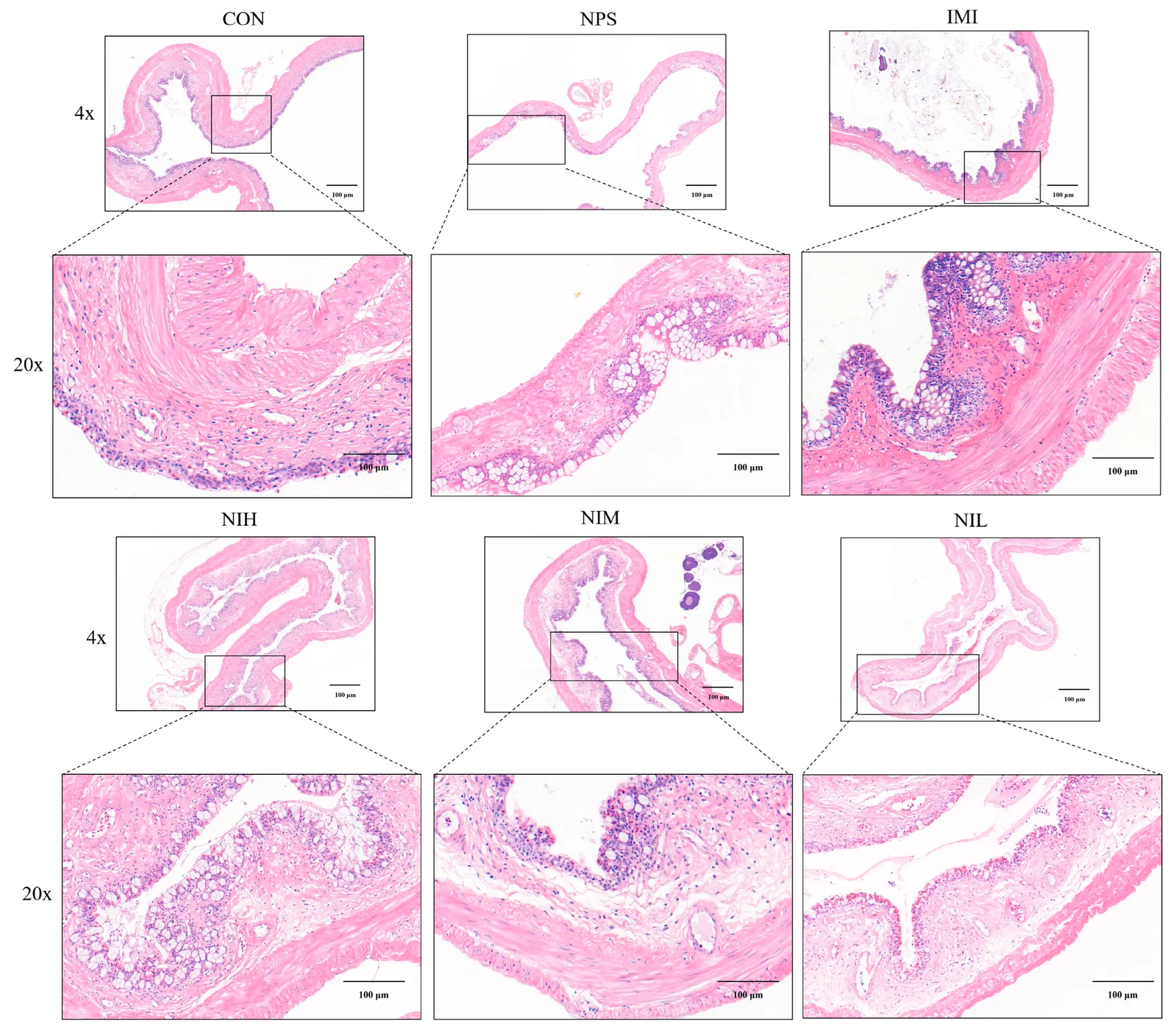
Representative hematoxylin-and-eosin-stained intestinal sections after 21 days of exposure. Low- and high-magnification views were obtained using 4× and 20× objectives, respectively. Boxes indicate enlarged regions. Exposure was associated with inflammatory infiltration, mucosal vacuolation, and disorganization of the intestinal wall; injury was most pronounced in NIH. Scale bars = 100 μm.

The intestinal wall of CON fish was continuous and organized, with preserved mucosal and submucosal architecture (Figure 5). Exposure groups exhibited inflammatory-cell accumulation, mucosal vacuolation, epithelial disorganization, and loosening at the mucosa–submucosa interface. Co-exposure produced more extensive abnormalities than IMI alone, and NIH showed the clearest loss of mucosal integrity.

**Figure 6:**
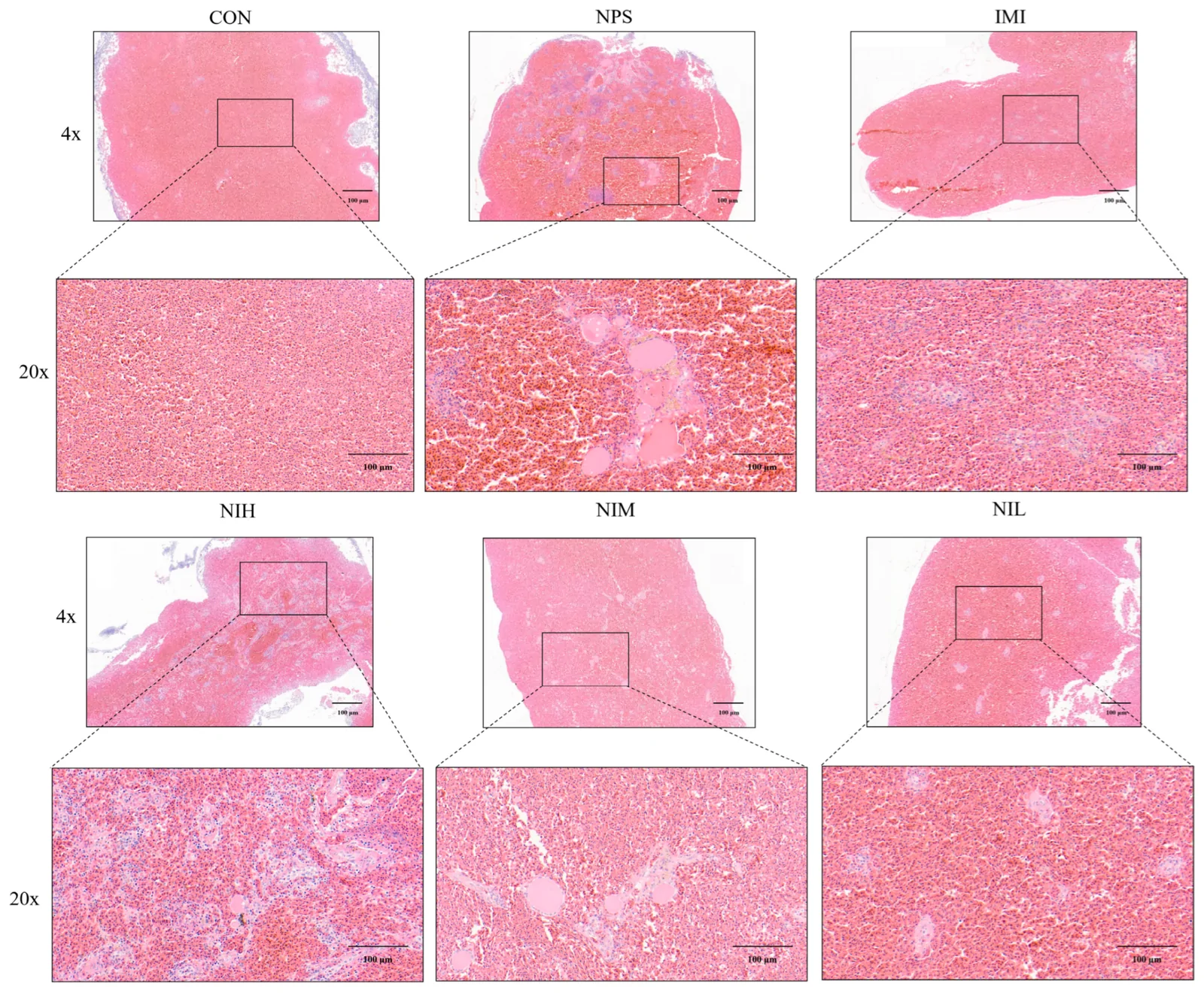
Representative hematoxylin-and-eosin-stained liver sections after 21 days of exposure. Low- and high-magnification views were obtained using 4× and 20× objectives, respectively. Boxes indicate enlarged regions. Exposure caused hepatocellular vacuolation, inflammatory infiltration, disorganization, and necrotic changes, with severe lesions in NIH. Scale bars = 100 μm.

Livers from the CON group showed compact and orderly hepatocyte organization (Figure 6). NPS and IMI alone produced variable vacuolation, inflammatory infiltration, and disruption of hepatic cords. Co-exposure increased tissue disorganization and necrotic change. The NIH group exhibited extensive inflammatory infiltration and loss of normal hepatic architecture, consistent with severe hepatic injury.

Histopathological lesion scores from four fish per treatment were summarized descriptively. The NIH group showed the highest fish-level lesion scores in the gill (3.00 ± 0.00), intestine (2.75 ± 0.50), and liver (2.75 ± 0.50), whereas the CON group showed no obvious histopathological lesions. The NIM group showed intermediate lesion severity, with the highest score observed in the gill. These fish-level scores were consistent with the qualitative microscopic observations and supported the greater histopathological injury observed in the high-level co-exposure group (Supplementary Table S3).

### 3.3. Overview of the Intestinal Transcriptome

**Figure 7:**
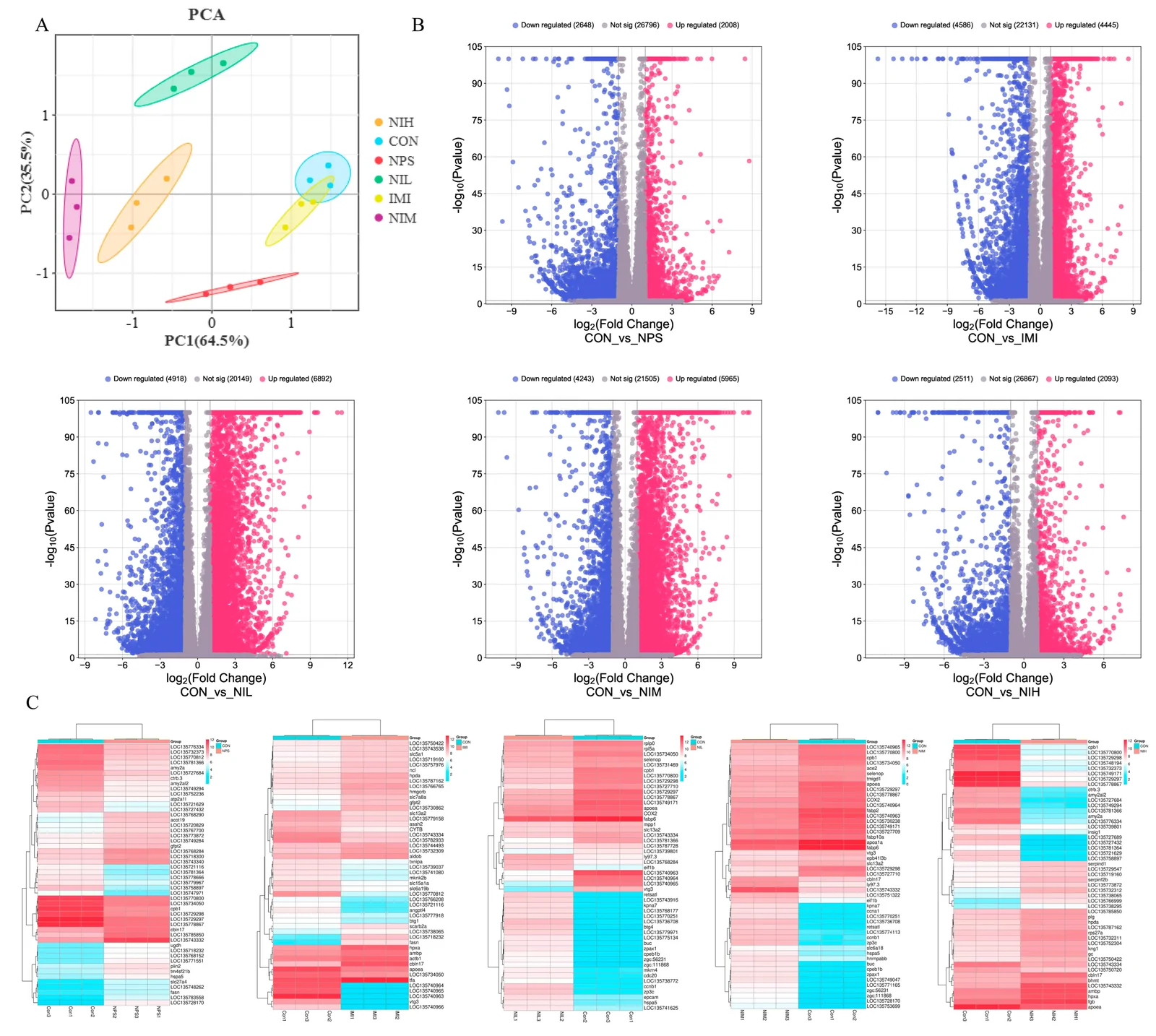
Global intestinal transcriptomic responses after Benjamini–Hochberg FDR correction. (**A**) Principal-component analysis of normalized gene-level count data from the six treatment groups (*n* = 3 biological replicates per treatment). Each point represents one biological replicate, and ellipses delineate the corresponding treatment-group distributions. (**B**) Volcano plots for CON versus NPS, IMI, NIL, NIM, and NIH. The *x*-axis represents log2(fold change), and the *y*-axis represents −log10(raw *p*). Pink and blue points indicate upregulated and downregulated genes, respectively, satisfying |log2(fold change)| > 1 and Benjamini–Hochberg-adjusted *p* < 0.05; gray points indicate genes that did not satisfy both criteria. Numbers in the legends indicate the corresponding gene counts. (**C**) Hierarchical clustering heatmaps of selected FDR-significant differentially expressed genes. Columns represent biological samples and rows represent genes. Red, white, and cyan indicate relatively high, intermediate, and low standardized expression, respectively.

RNA sequencing generated high-quality data, with a mean clean-read proportion of 98.93%, mean Q20 of 99.11%, mean Q30 of 97.35%, and mean GC content of approximately 44.8% (Table 3). Across the 18 libraries, the mean number of clean reads was 47.35 million per sample, and the mean mapping rate was 89.45%, with individual-library mapping rates ranging from 80.70% to 90.79%. Principal-component analysis separated most treatment groups and showed generally consistent clustering of biological replicates (Figure 7A).

Using |log2(fold change)| > 1 and Benjamini–Hochberg-adjusted *p* < 0.05, 4656 differentially expressed genes were detected in CON versus NPS (2008 upregulated and 2648 downregulated), 9031 in CON versus IMI (4445 upregulated and 4586 downregulated), 11,810 in CON versus NIL (6892 upregulated and 4918 downregulated), 10,208 in CON versus NIM (5965 upregulated and 4243 downregulated), and 4604 in CON versus NIH (2093 upregulated and 2511 downregulated) (Figure 7B and Table 4). Hierarchical clustering of selected FDR-significant genes separated the control and corresponding exposure samples (Figure 7C). NIL and NIM produced the largest FDR-controlled DEG sets, whereas NIH showed a smaller transcriptional response with only a modest predominance of downregulated genes. Thus, the corrected NIH comparison does not support the previously reported inference of widespread transcriptional repression.

### 3.4. GO and KEGG Enrichment of Differentially Expressed Genes

**Figure 8:**
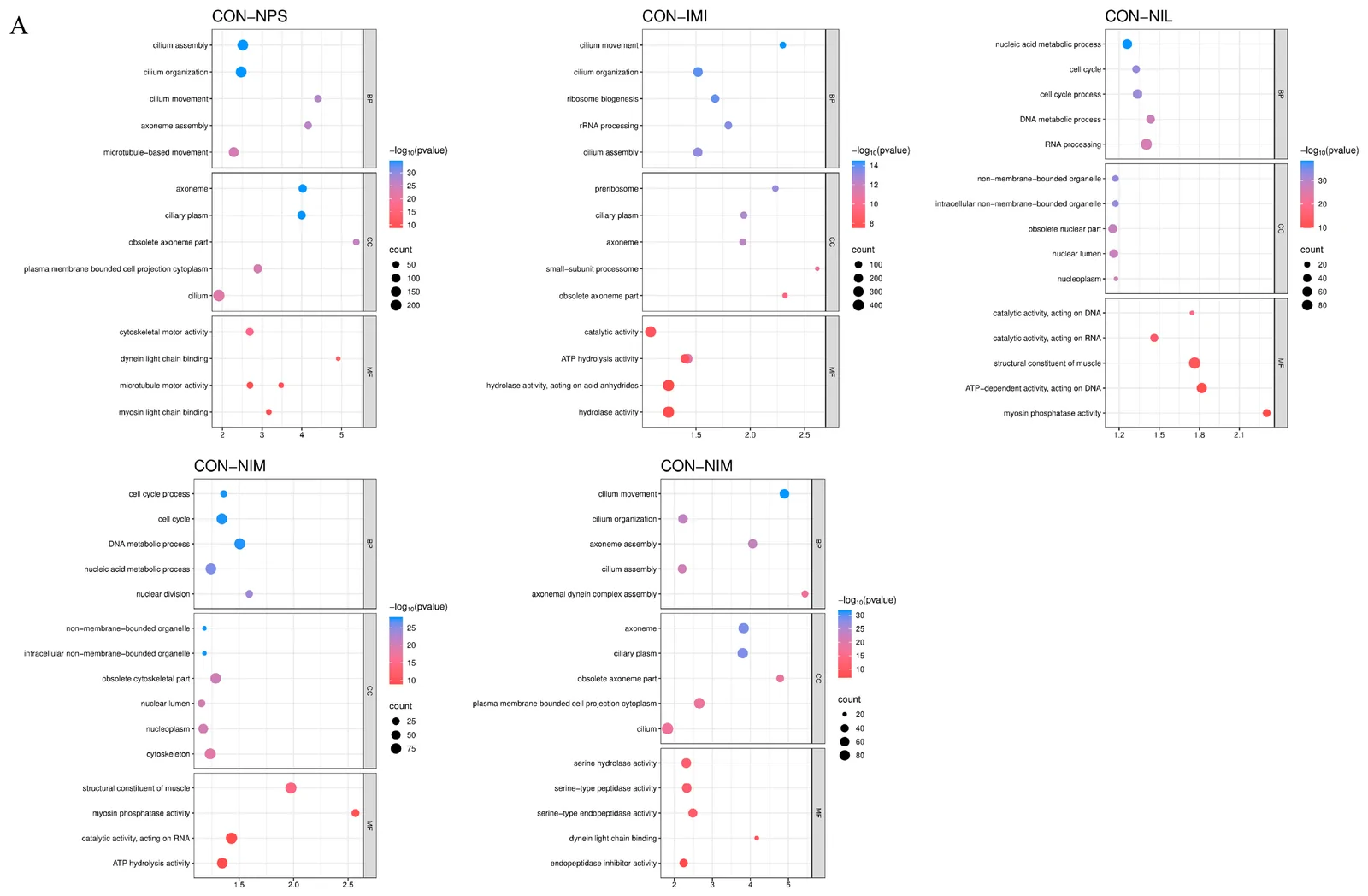

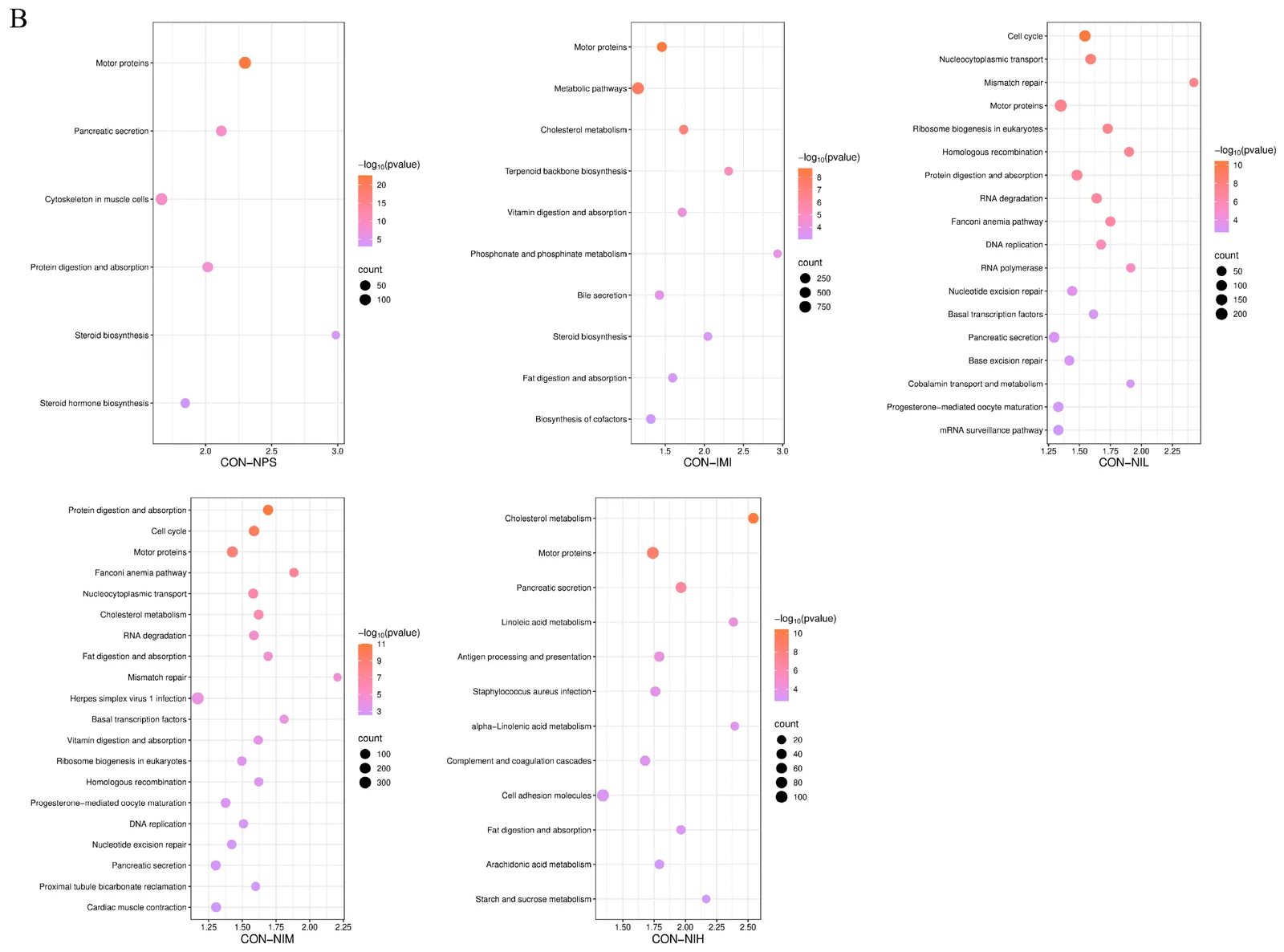
FDR-controlled functional enrichment of intestinal differentially expressed genes. (**A**) Gene Ontology enrichment for CON versus NPS, IMI, NIL, NIM, and NIH. The biological-process (BP), cellular-component (CC), and molecular-function (MF) domains are displayed for each comparison. (**B**) Benjamini–Hochberg FDR-significant Kyoto Encyclopedia of Genes and Genomes pathways for the same five comparisons. In both panels, the *x*-axis represents fold enrichment, bubble size represents the number of differentially expressed genes assigned to the term or pathway, and bubble color represents −log10(raw *p*). Only terms or pathways satisfying Benjamini–Hochberg-adjusted *p* < 0.05 are displayed.

Following Benjamini–Hochberg correction, Gene Ontology enrichment revealed comparison-specific functional responses across the biological-process, cellular-component, and molecular-function domains (Figure 8A). Terms related to cilium assembly, organization, and movement, axoneme structure, ciliary components, and microtubule-associated motor activity were prominent in the NPS, IMI, and NIH comparisons. In contrast, the NIL and NIM comparisons were characterized primarily by cell-cycle progression, nuclear division, nucleic-acid and DNA metabolic processes, and RNA-associated functions. Catalytic, ATP-hydrolysis, hydrolase, peptidase, cytoskeletal-motor, and structural-muscle activities were also represented among the enriched molecular functions. These findings indicate that the principal functional responses differed among exposure conditions rather than following a uniform pattern across all comparisons.

After Benjamini–Hochberg correction, 6, 11, 19, 21, and 13 KEGG pathways were significantly enriched in CON versus NPS, IMI, NIL, NIM, and NIH, respectively (Figure 8B). The NPS comparison was characterized by motor proteins, pancreatic secretion, cytoskeletal organization, protein digestion and absorption, steroid biosynthesis, and steroid-hormone biosynthesis. The IMI comparison included metabolic pathways, cholesterol metabolism, bile secretion, steroid biosynthesis, and vitamin and fat digestion and absorption. NIL and NIM showed prominent enrichment of cell-cycle, DNA-repair, transcriptional, cytoskeletal, and digestive pathways; cholesterol metabolism and fat digestion and absorption were additionally significant in NIM. The NIH comparison included protein digestion and absorption, cholesterol metabolism, pancreatic secretion, fat digestion and absorption, and linoleic-, alpha-linolenic-, and arachidonic-acid metabolism, together with several immune-related pathways. These FDR-controlled results support treatment-associated disturbances of intestinal digestive, proliferative, and lipid-homeostatic processes, although the specific pathway pattern differed among comparisons.

### 3.5. Changes in the Intestinal Metabolic Features

**Figure 9:**
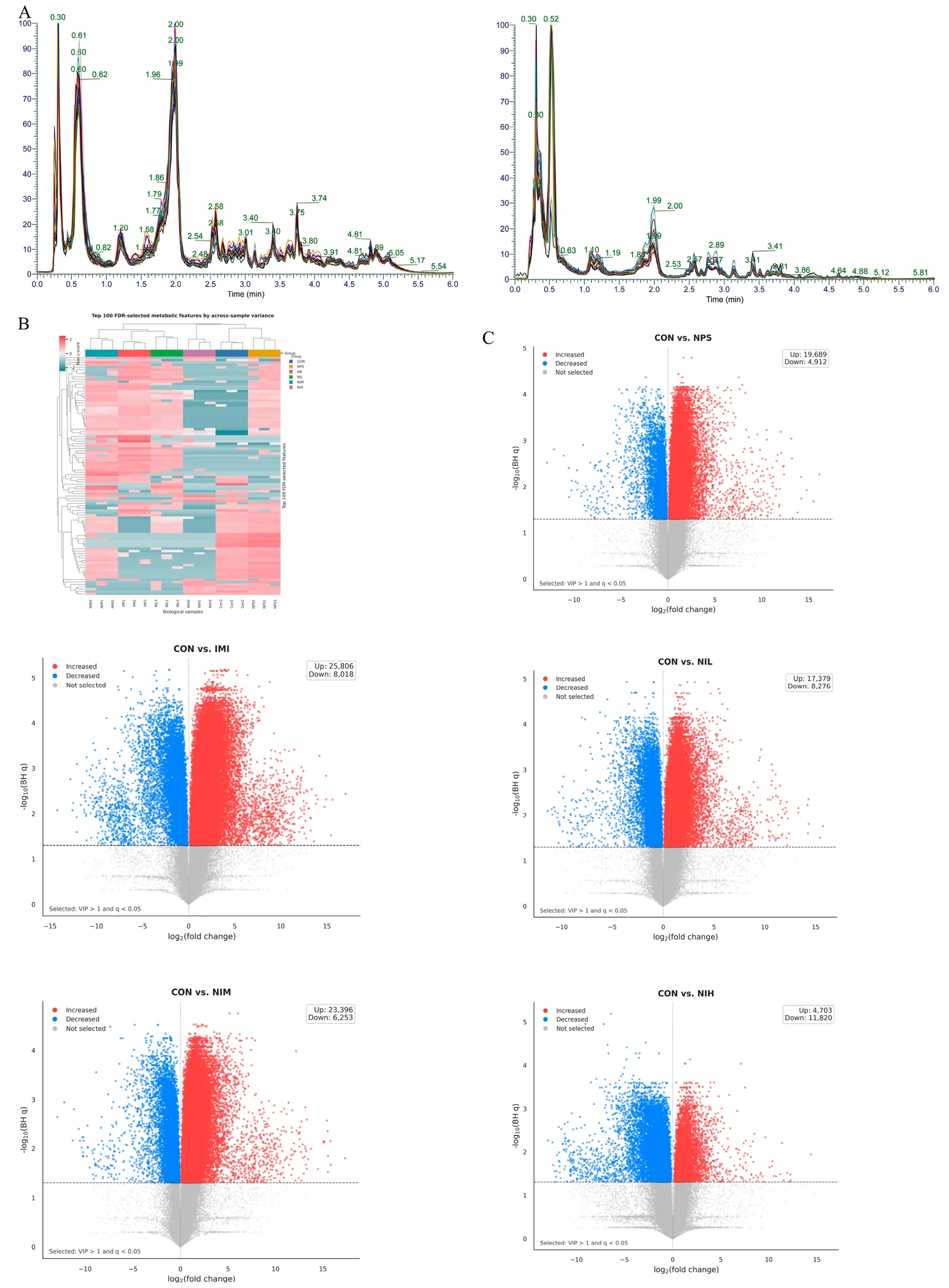

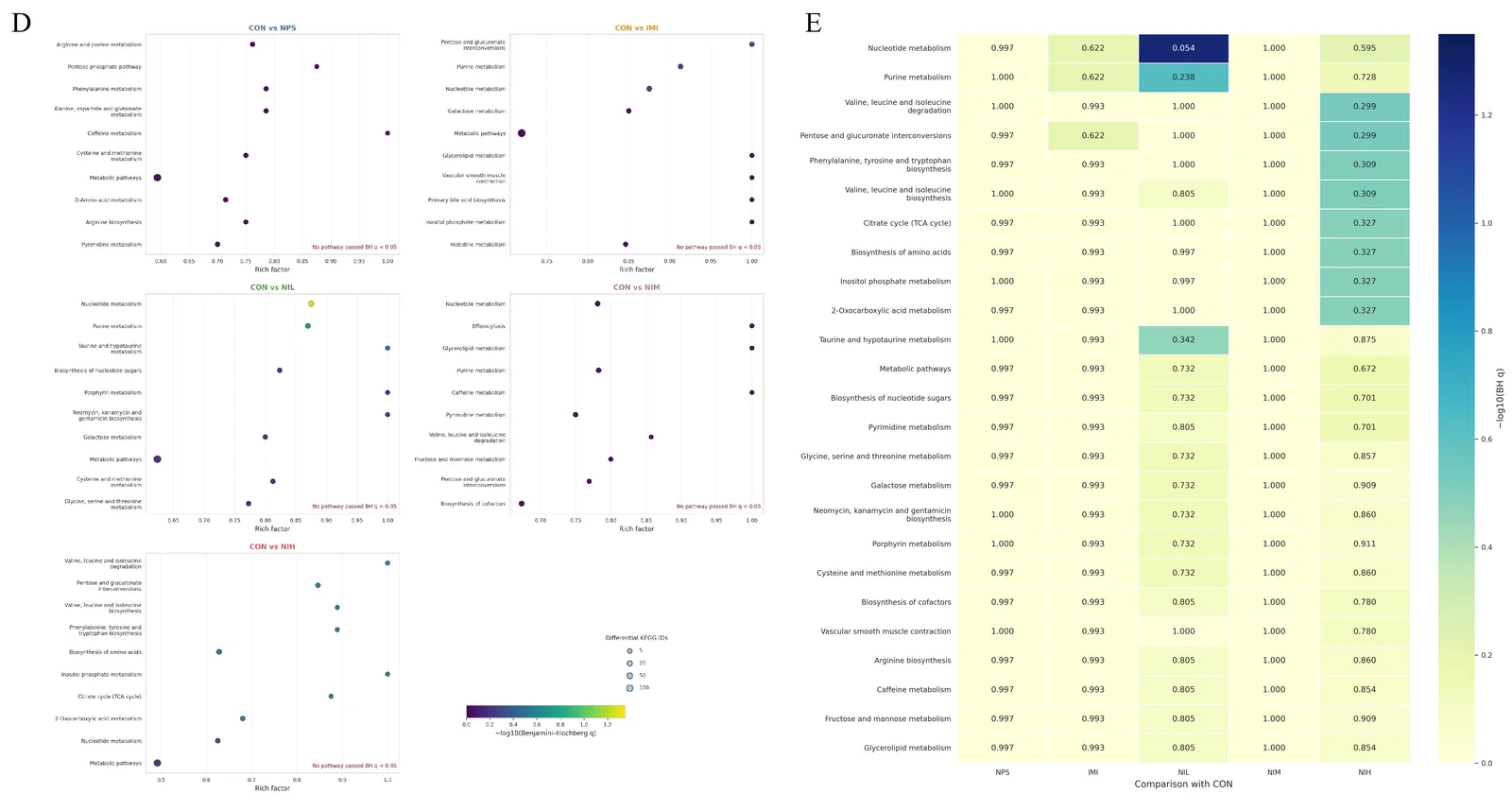
FDR-controlled intestinal metabolomic responses under single- and co-exposure conditions. (**A**) Overlaid total-ion chromatograms of pooled quality-control samples in positive- and negative-ionization modes. (**B**) Hierarchical clustering heatmap of the 100 unique FDR-selected peaks with the greatest variance across the 18 biological samples after log2(intensity + 1) transformation and row z-score standardization. (**C**) Volcano plots for CON versus NPS, IMI, NIL, NIM, and NIH. The *x*-axis represents log2(fold change), and the *y*-axis represents −log10(Benjamini–Hochberg q). Red and blue indicate increased and decreased feature records satisfying VIP > 1 and q < 0.05, respectively; gray indicates records not satisfying both criteria. The horizontal dashed line denotes q = 0.05. (**D**) KEGG pathway over-representation results showing the 10 top-ranked pathways in each comparison. The *x*-axis represents the rich factor, bubble size represents the number of unique differential KEGG compound identifiers, and bubble color represents −log10(BH q). (**E**) Heatmap of metabolomic pathway BH q values across the five comparisons. No metabolomic pathway met the prespecified q < 0.05 threshold.

The total-ion chromatograms of the pooled quality-control samples showed good overlap in both positive- and negative-ionization modes, indicating stable LC–MS performance throughout the analytical sequence (Figure 9A). Hierarchical clustering of the 100 unique FDR-selected peaks with the greatest across-sample variance grouped the three biological replicates from each treatment together (Figure 9B).

Using the criteria of VIP > 1 and Benjamini–Hochberg q < 0.05, 24,601 differential feature records were identified for CON versus NPS, including 19,689 increased and 4912 decreased records. Correspondingly, 33,824 records were identified for CON versus IMI (25,806 increased and 8018 decreased), 25,655 for CON versus NIL (17,379 increased and 8276 decreased), 29,649 for CON versus NIM (23,396 increased and 6253 decreased), and 16,523 for CON versus NIH (4703 increased and 11,820 decreased) (Figure 9C and Table 5). Increased peaks predominated in the NPS, IMI, NIL, and NIM comparisons, whereas decreased peaks predominated in the NIH comparison.

Among the FDR-selected records, 2042, 2440, 2030, 2214, and 1415 records had MS/MS library matches in BiotreeDB v3.0 for the NPS, IMI, NIL, NIM, and NIH comparisons, respectively. After removal of duplicate records, these corresponded to 1967, 2353, 1958, 2140, and 1344 unique Level 2 putatively annotated peaks, respectively.

KEGG pathway mapping highlighted sphingolipid metabolism, glycerophospholipid metabolism, biosynthesis of unsaturated fatty acids, fatty acid elongation, and glycerolipid metabolism (Figure 9D,E). The pathway profiles included carbohydrate-, amino-acid-, nucleotide-, and lipid-related processes.

### 3.6. Exploratory Correlation Analysis and qPCR Validation

**Figure 10:**
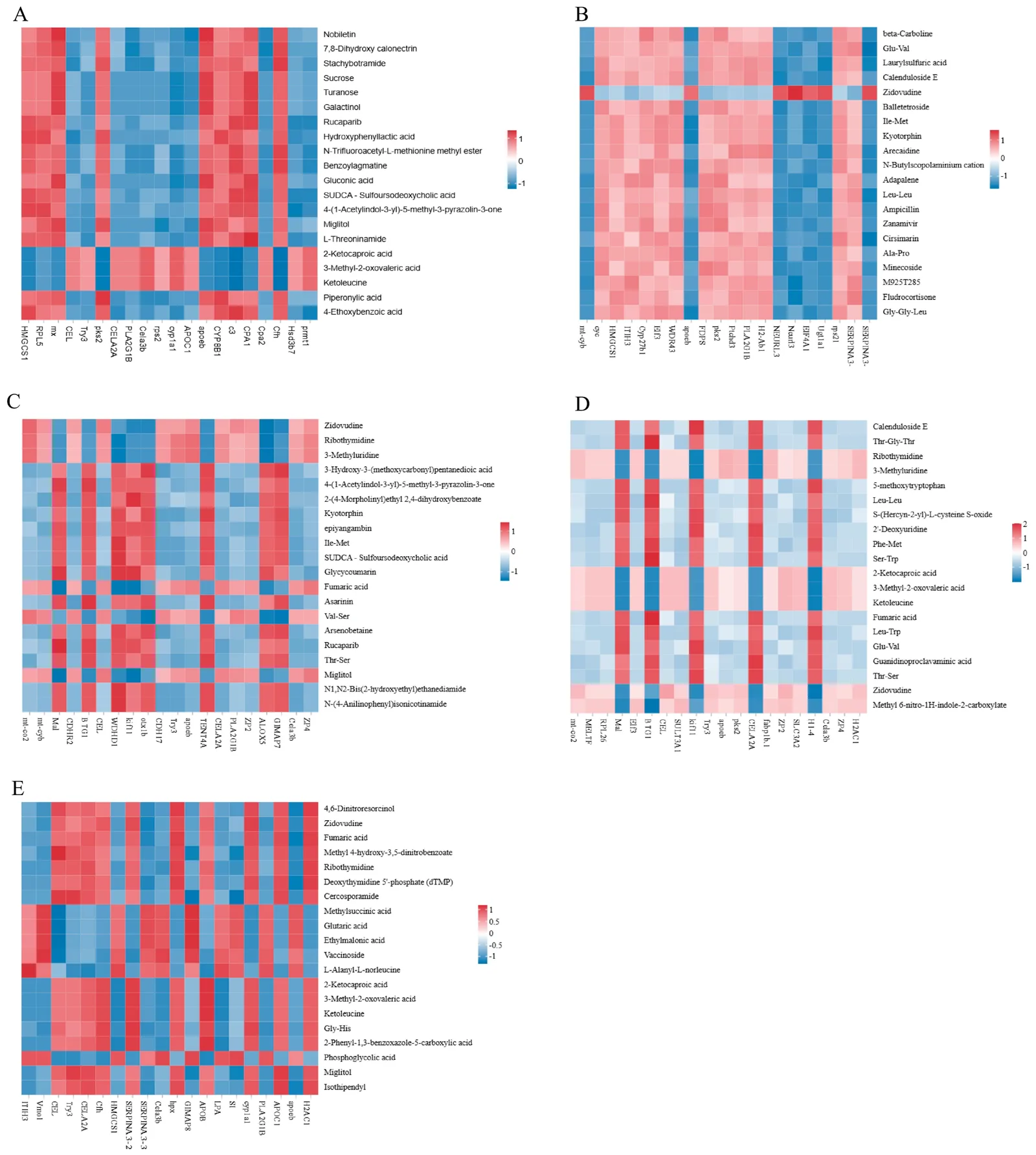
Exploratory correlations between selected intestinal transcripts and metabolic features. (**A**) CON versus NPS. (**B**) CON versus IMI. (**C**) CON versus NIL. (**D**) CON versus NIM. (**E**) CON versus NIH. Pearson correlation coefficients were calculated using matched normalized gene-expression and metabolic-feature-intensity data from the control and corresponding exposure samples. Red and blue indicate positive and negative correlations, respectively, with greater color intensity representing a larger absolute correlation coefficient.

Exploratory correlation heatmaps showed comparison-specific relationships between selected FDR-significant transcripts and FDR-selected, MS2-annotated metabolic features (Figure 10). Frequently represented genes included *HMGCS1*, *CYP8B1*, *CEL*, *APOB*, *APOC1*, *CYP27B1*, *CFH*, *CELA2A*, *GIMAP8*, *BTG1*, and *NEURL3*. The correlated feature annotations included carbohydrates, amino acids and dipeptides, organic acids, bile-acid-related compounds, and lipid-associated molecules. Because the correlations were calculated from a limited number of matched biological samples and numerous gene–feature pairs were examined, these patterns were interpreted as exploratory associations rather than independently significant gene–metabolite relationships.

**Figure 11:**
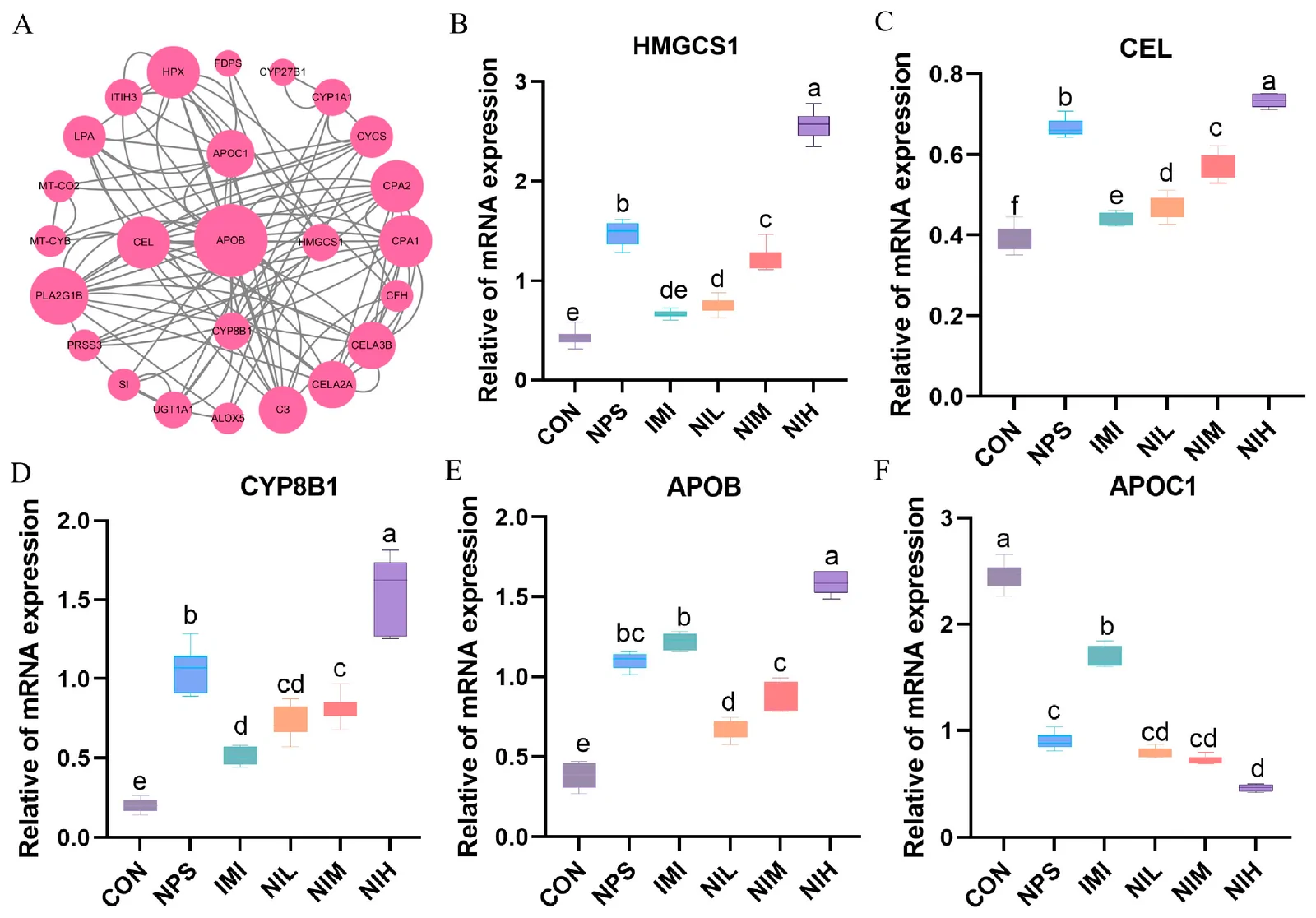
Protein-interaction analysis and quantitative PCR validation of lipid-related genes. (**A**) Protein–protein interaction network; larger nodes indicate greater connectivity. Relative mRNA expression of (**B**) *HMGCS1*, (**C**) *CEL*, (**D**) *CYP8B1*, (**E**) *APOB*, and (**F**) *APOC1*. Data are shown as box-and-whisker plots. Groups with different lowercase letters differ significantly (*p* < 0.05).

The protein–protein interaction network placed APOB at the center of a lipid-related module that included *HMGCS1*, *CEL*, *CYP8B1*, and *APOC1* (Figure 11A). Quantitative PCR confirmed significant treatment-associated changes in all five genes (Figure 11B–F). *HMGCS1*, *CEL*, and *CYP8B1* generally showed higher expression in the exposure groups and reached high levels in NIH. APOB exhibited a non-linear response across the co-exposure groups: its expression in NIL was lower than that in the NPS and IMI groups, followed by an increase at the higher PS-NP co-exposure levels, with the highest expression observed in NIH. APOC1 showed an overall decreasing pattern and reached its lowest level in NIH. These gene-specific expression profiles independently support treatment-associated changes in intestinal cholesterol synthesis, bile-acid regulation, lipid digestion, and lipoprotein transport.

## 4. Discussion

Both imidacloprid (IMI) and polystyrene nanoplastics (PS-NPs) can enter freshwater environments and may coexist in water and sediments, thereby posing a co-exposure risk to the loach *Misgurnus anguillicaudatus*, a benthic freshwater fish. Previous studies have shown that IMI can cause oxidative stress, inflammation, DNA damage, intestinal barrier disruption and metabolic disorders in fish [24,25,26]. Due to their small particle size, PS-NPs are easily taken up by fish and distributed in different tissues, and their toxicity is mainly manifested as decreased antioxidant capacity, enhanced inflammatory response, and imbalance of energy and lipid metabolism [27,28]. In addition, plastic particles have a large specific surface area that can adsorb coexisting organic pollutants and may alter the migration, bioavailability, and tissue distribution of these pollutants [29]. Su et al. [23] reported that IMI exposure caused intestinal morphological injury, impaired antioxidant capacity, enhanced inflammatory responses, and altered intestinal metabolism in loaches. In the present study, both PS-NP and IMI single exposure were associated with adverse responses, whereas the co-exposure groups showed lower survival and stronger oxidative-stress, inflammatory, and histopathological responses, particularly at the higher PS-NP concentrations within the co-exposure series. These findings indicate that PS-NP co-exposure was associated with stronger IMI-related adverse responses under the tested conditions. However, because PS-NP-only groups at 25 and 50 μg/L were not included, these observations should be interpreted as an exposure-gradient pattern within the co-exposure series rather than as formal evidence of a synergistic interaction.

Oxidative stress and inflammatory response may be an important basis for multiple organ damage caused by combined exposure [30,31]. CAT and SOD are key antioxidant enzymes involved in limiting oxidative damage, whereas GSH contributes to cellular redox homeostasis [32,33]. MDA is an important product of lipid peroxidation [34]. In this study, CAT, SOD and GSH levels decreased, while MDA level increased after exposure, and the changes were most obvious in the NIH group, indicating impaired antioxidant capacity and enhanced lipid peroxidation under high-level co-exposure. At the same time, the expression of pro-inflammatory factors *IL-1β*, *IL-6*, *IL-8* and *TNF-α* increased, and the expression of anti-inflammatory factors *IL-4* and *IL-10* decreased, indicating that the liver immune response changed from a relatively balanced state to a pro-inflammatory state [35]. IMI has been shown to induce oxidative stress and inflammation in grass carp hepatocytes through NF-κB/JNK and other pathways [36], and PS-NPs can also activate the inflammatory response related to NF-κB/NLRP3 [37]. Therefore, the inflammatory changes observed in this study may be related to persistently enhanced oxidative stress: excess reactive oxygen species are able to initiate inflammatory signals, which in turn may further promote oxidative damage, forming a mutually amplifying process [38]. Li et al. [39] observed PS-NP-induced oxidative damage in the liver of large yellow croaker, while Ling et al. [40] reported that PS-NPs enhanced microcystin-LR uptake and exacerbated oxidative liver damage in zebrafish. These observations are consistent with the oxidative-stress pattern observed in the present study.

Gills are the main organs of aquatic animals that are directly exposed to water pollutants, while intestine is affected by both water intake and food exposure [41]. Liver is an important site for pollutant transformation and nutrient metabolism [42]. In the present study, gill filament bending, epithelial hyperplasia or fusion, intestinal mucosal structure disorder and hepatic cell vacuolization were caused by single exposure. After co-exposure, the structural damage of gill, intestinal mucosal injury, inflammatory cell infiltration and liver tissue disorder were further aggravated, and the damage in the NIH group was the most obvious. The results suggest that the effects of co-exposure are not limited to a single organ, but involve multiple tissues related to contaminant exposure, absorption, and metabolism. PS-NPs have been reported to disturb intestinal homeostasis in fish [6,43], while IMI has been shown to increase intestinal permeability and disrupt tight-junction integrity in other fish models [44]. In the present study, histopathological examination demonstrated intestinal mucosal injury; however, intestinal permeability and tight-junction function were not directly measured. Therefore, altered gut–liver communication involving inflammatory mediators or metabolites remains a plausible explanation for the concurrent intestinal and hepatic responses rather than a mechanism directly demonstrated here.

Transcriptomic analysis further characterized treatment-associated changes in intestinal function. After FDR correction, NIL and NIM yielded the largest DEG sets, whereas NIH yielded a smaller DEG set with only a modest predominance of downregulated genes. Therefore, the corrected NIH comparison did not support widespread transcriptional repression. FDR-significant GO terms related to the cell cycle, chromosome segregation, cilia, and microtubules indicated treatment-associated changes in processes involved in intestinal epithelial renewal and structural maintenance. FDR-significant KEGG pathways included protein digestion and absorption, vitamin digestion and absorption, pancreatic secretion, fat digestion and absorption, and cholesterol metabolism, supporting disturbances in intestinal digestive and lipid-homeostatic processes. Gonzalez-Acedo et al. [45] similarly summarized the adverse effects of micro- and nanoplastics on the digestive system. The present findings further suggest that IMI exposure was associated with altered intestinal digestive processes and that the response patterns differed among the co-exposure conditions.

After FDR correction, the numbers and directions of differentially expressed genes varied among comparisons. NIL and NIM yielded the largest FDR-controlled DEG sets, whereas NIH yielded a smaller DEG set with only a modest predominance of downregulated genes. At the metabolomic feature level, increased FDR-selected feature records predominated in the NPS, IMI, NIL, and NIM comparisons, whereas decreased records predominated in NIH. At the metabolomic feature level, increased FDR-selected feature records predominated in the NPS, IMI, NIL, and NIM comparisons, whereas decreased records predominated in NIH. KEGG pathway mapping highlighted sphingolipid metabolism, glycerophospholipid metabolism, biosynthesis of unsaturated fatty acids, fatty acid elongation, and glycerolipid metabolism. Because sphingolipids and glycerophospholipids are major components of cell membranes and participate in inflammatory signaling, alterations in these pathways may be associated with membrane lipid remodeling, intestinal mucosal injury, and inflammatory responses. Features putatively assigned to hydroxyphenyllactic acid, deaminated tyrosine, and miglitol showed altered intensities in selected comparisons. These assignments were based on MS/MS library matches in BiotreeDB v3.0 but were not confirmed using authentic standards; they were therefore classified as Level 2 putative annotations rather than confirmed metabolite identities. Compounds corresponding to these putative annotations have previously been associated with intestinal homeostasis, redox and inflammatory responses [46,47], and glucose or lipid metabolism [48]. However, the present findings should be regarded as exploratory and do not establish that these specific compounds were mechanistically involved in the intestinal injury associated with IMI and PS-NP exposure.

Lipid metabolism was the main direction of change that both transcriptome and qPCR results pointed to. In the present study, HMGCS1, CEL, and CYP8B1 generally increased under exposure, APOB showed a non-linear response across the co-exposure groups, and APOC1 showed an overall decreasing trend. HMGCS1 is involved in the mevalonate pathway and is an important regulator of cholesterol synthesis [49,50]. Its elevation may reflect a compensatory response to membrane lipid damage or disruption of cholesterol [51,52]. CEL is involved in intestinal lipid hydrolysis and lipoprotein metabolism [53,54], while APOB is a structural protein that is essential for the assembly and transport of triglyceride-rich lipoproteins [55]. Changes in CEL and APOB expression suggest remodeling of intestinal lipid digestion and lipoprotein transport. CYP8B1 is involved in the regulation of bile acid composition and can affect intestinal fat absorption and cholesterol homeostasis [56,57]. The altered CYP8B1 expression is consistent with disruption of bile-acid-related and intestinal lipid metabolic processes. APOC1 regulates lipoprotein lipase activity and residual lipoprotein clearance [58]; APOC1 reduction may further perturb lipoprotein remodeling and lipid clearance. The changes in the above genes did not prove lipid accumulation alone, but supported each other by the enrichment results of cholesterol metabolism and fat digestion and absorption in the transcriptome, suggesting that combined exposure changed the balance between lipid synthesis, digestion, transport, and clearance. Previous studies have observed abnormal lipid metabolism caused by PS-NPs in large yellow croaker, zebrafish, and mice [59,60], consistent with the results of this study. Importantly, the qPCR-validated genes and the metabolomic pathways do not represent a simple one-to-one gene–metabolite relationship, but they converge on a broader disturbance of intestinal lipid homeostasis. Elevated HMGCS1 expression suggests altered cholesterol synthesis, whereas changes in CEL, APOB, and APOC1 indicate remodeling of lipid digestion and lipoprotein transport. CYP8B1 further linked these responses to bile acid composition as well as intestinal lipid absorption. At the same time, metabolomic pathway mapping highlighted glycerophospholipid and sphingolipid metabolism, two pathways related to membrane lipid turnover and lipid-mediated signaling. Therefore, the transcriptomic, qPCR, and metabolomic results consistently indicated disturbances in lipid synthesis, digestion, transport, and membrane lipid composition, providing mutually supportive evidence that lipid metabolic dysregulation is a major feature of the intestinal response to combined PS-NP and IMI exposure.

Taken together, the present findings support a progressive interpretation of the combined toxicity while distinguishing the measured responses from the proposed mechanisms. Polystyrene nanoparticles (PS-NPs) have a large specific surface area and are able to interact with coexisting organic pollutants. Previous studies have shown that nanoscale plastic particles can alter the adsorption behavior of pollutants and their uptake by organisms. Therefore, PS-NPs may have altered the effective exposure level of imidacloprid (IMI) in the present study system, although IMI adsorption on PS-NPs and IMI load in tissues were not directly measured. In this exposure context, co-exposed fish exhibited a progressive decrease in antioxidant capacity, as indicated by decreased CAT, SOD, and GSH activities accompanied by increased MDA levels, suggesting enhanced oxidative stress and lipid peroxidation. These changes were also accompanied by up-regulation of pro-inflammatory gene expression and down-regulation of anti-inflammatory gene expression. Thus, oxidative stress and inflammatory signaling pathways may act in concert to contribute to the histologically observed intestinal mucosal injury. At the molecular level, transcriptome and metabolome analysis further revealed disturbances in digestive function and lipid homeostasis, including abnormal metabolism of cholesterol, bile acids, glycerophospholipids, and sphingolipids. These molecular perturbations are consistent with lipid-related gene-expression changes validated by qPCR and may collectively contribute to the broader tissue injury and reduced survival observed under co-exposure.

An important limitation of the present study is that the physicochemical behavior of PS-NPs in the exposure water was not monitored during the 21-day experiment. Changes in particle aggregation, sedimentation, hydrodynamic size, and surface charge may therefore have affected the actual exposure experienced by the fish. In addition, dissolved and particle-associated IMI concentrations were not measured, and thus any PS-NP-mediated alteration in IMI distribution or bioavailability could not be directly demonstrated. Accordingly, this possible PS-NP-mediated alteration in IMI exposure should be regarded as a plausible interpretation supported by previous studies rather than as a mechanism directly confirmed here.

In addition, because PS-NP-only groups at 25 and 50 μg/L were not included, the present design does not allow formal assessment of concentration-dependent effects of PS-NPs alone or statistical interaction between PS-NPs and IMI. Therefore, the stronger responses observed across the NIL, NIM, and NIH groups should be interpreted as an exposure-gradient trend within the co-exposure series rather than evidence of a formally demonstrated dose–response or synergistic interaction.

Future studies should combine time-resolved particle characterization with analytical determination of dissolved and particle-associated IMI and a full factorial exposure design to better distinguish concentration-related effects from PS-NP × IMI interactions.

## 5. Conclusions

Under the tested conditions, co-exposure to IMI and PS-NPs produced broader adverse responses than IMI exposure alone, although the present experimental design does not permit formal determination of synergistic or additive interactions. Increasing PS-NP concentrations within the co-exposure series were associated with stronger disturbances in hepatic oxidative balance, inflammatory responses, and histopathological injury of the gill, intestine, and liver. Intestinal transcriptomic and metabolomic analyses further showed that these tissue-level effects were accompanied by disruption of digestive processes, cholesterol and bile-acid metabolism, glycerophospholipid and sphingolipid metabolism, and other lipid-related pathways. In the NIH group, decreased metabolic features predominated, whereas the transcriptomic DEG set showed only a modest excess of downregulated genes, indicating distinct response patterns between the two omics datasets under high-level co-exposure.

The integrated results indicate that the biological consequences of PS-NP and IMI co-exposure extend beyond localized oxidative stress and inflammation to involve multi-organ injury and substantial disturbance of intestinal metabolic homeostasis. These findings have implications for environmental risk assessment because aquatic organisms are exposed to complex contaminant mixtures rather than isolated chemicals, and risk estimates based solely on single-contaminant toxicity may not fully capture the biological responses that occur when pesticides coexist with nanoplastic pollution in freshwater habitats. Nevertheless, the exposure concentrations used in this study were experimental sublethal concentrations and should not be directly extrapolated to environmentally realistic exposure scenarios.

Future studies should therefore incorporate environmentally relevant concentration ranges, full factorial exposure designs, time-resolved characterization of PS-NP aggregation and surface properties, analytical verification of dissolved and particle-associated IMI, particle–pollutant binding and desorption kinetics, and tissue-specific bioaccumulation measurements. Field or mesocosm validation will also be important for determining whether the exposure-response patterns observed here occur under more complex environmental conditions.

## Figures and Tables

**Table 1:** Experimental groups and exposure concentrations.

Group	PS-NPs (μg/L)	IMI (μg/L)	Description
CON	0	0	Clean-water control
NPS	100	0	PS-NPs alone
IMI	0	78	IMI alone
NIL	25	78	Low PS-NP co-exposure
NIM	50	78	Medium PS-NP co-exposure
NIH	100	78	High PS-NP co-exposure

Note: Each treatment contained three replicate tanks with 30 fish per tank (90 fish per treatment).

**Table 2:** Primer sequences used for quantitative real-time PCR.

Gene	GenBank Accession no.	Forward Primer (5′–3′)	Reverse Primer (5′–3′)	Product (bp)
*il4*	XM_073812116.1	TGGAGGAAACTATGGAGA	GACAGCGTAGGAAGAAAG	194
*il10*	XM_065242112.1	CTCTGCTCACGCTTCT	TCCATAGGGACTGTTTAT	183
*il8*	XM_064337872.1	AGAGCCTTGAGGGAGTA	GTGGGAGTTGGGTGGAA	158
*il1b*	XM_065257802.1	CGCATCCTCACAGCATGAA	ACGTAAGACTGAGCTGCCC	295
*tnfa*	XM_065294432.1	GGTACCAGGATCAGGCGTTC	TATCGCATCACTGCATGGCT	181
*il6*	XM_065284638.2	ATTGGAGGTCTTGGTGGA	GGATTGGTTGGAGGGTCTA	322
*hmgcs1*	XM_073816085.1	CTACACCTGGGTCTGCC	CAATGAAACCTGCCTCC	162
*cel*	XM_027004233.3	CACTGAAGGAGGGATGGT	AACAGGCAAGTTGGAGGA	154
*cyp8b1*	XM_035428079.1	AGCCGTTCTTCAGGATA	GAATGGCAGTGTAGGGA	186
*apob*	XM_031866816.1	CGATTCCTCACCCAGAT	CCATACGCACCTTCACA	179
*apoc1*	XM_065260991.2	CACAGAAGCGGAAATGAT	CGTGGCAGGAGTGAGAA	133
*gapdh*	XM_065270132.2	ACCGTGTCTGCGACCTGA	AGCTATGACTACCGAT	291

**Table 3:** Summary of intestinal RNA-sequencing quality metrics.

Group	Clean Reads (%)	GC (%)	Q20 (%)	Q30 (%)
CON	98.99	44.80	99.13	97.38
NPS	98.99	44.41	99.15	97.46
IMI	98.96	44.70	99.12	97.36
NIL	98.69	45.79	98.98	97.02
NIM	98.98	44.77	99.15	97.45
NIH	98.96	44.25	99.14	97.44

Values are group means calculated from three biological RNA-seq libraries per treatment (*n* = 3).

**Table 4:** Differentially expressed genes relative to the control group.

Comparison	Upregulated	Downregulated	Total
CON vs. NPS	2008	2648	4656
CON vs. IMI	4445	4586	9031
CON vs. NIL	6892	4918	11,810
CON vs. NIM	5965	4243	10,208
CON vs. NIH	2093	2511	4604

Differentially expressed genes were defined by |log2(fold change)| > 1 and Benjamini–Hochberg-adjusted *p* < 0.05.

**Table 5:** Differential intestinal LC–MS features and Level 2 putatively annotated peaks.

Comparison	Differential Feature Records	MS/MS-Annotated Records	Unique Level 2 Putatively Annotated Peaks
CON-NPS	24,601	2042	1967
CON-IMI	33,824	2440	2353
CON-NIL	25,655	2030	1958
CON-NIM	29,649	2214	2140
CON-NIH	16,523	1415	1344

Differential feature records were selected using VIP > 1 and Benjamini–Hochberg q < 0.05. Unique Level 2 putatively annotated peaks were obtained by removing exact duplicate MS/MS-matched records sharing ion mode, *m*/*z*, retention time, and all 18 normalized biological-sample intensities. Different adducts, isotopes, and positive/negative-ion records were not merged because validated peak-grouping information was unavailable. No metabolite met the MSI Level 1 identification criteria because authentic-standard confirmation was unavailable.

## Data Availability

The original data presented in this study are openly available in Mendeley Data at https://doi.org/10.17632/mfgygmpwmy.1.

## Supplementary Materials

The following supporting information can be downloaded at: https://www.mdpi.com/article/10.3390/biology15181608/s1, Supplementary Table S1: Detailed statistical analyses of survival and hepatic oxidative-stress biomarkers; Supplementary Table S2: Amplification efficiency and linearity of qPCR assays; Supplementary Table S3: Fish-level semi-quantitative histopathological lesion scores in the gill, intestine, and liver (*n* = 4 fish per treatment). Supplementary Table S4: Full differential LC–MS feature tables containing feature ID, ion mode, m/z, retention time, fold change, raw *p* value, BH q value, VIP score, annotation, and MSI confidence level. File S1: Raw data.

## Abbreviations

The following abbreviations are used in this manuscript:

AMPK	AMP-activated protein kinase
APOB	Apolipoprotein B
APOC1	Apolipoprotein C1
CAT	Catalase
CEL	Carboxyl ester lipase
CON	Control group
CYP8B1	Cytochrome P450 family 8 subfamily B member 1
DEGs	Differentially expressed genes
ESI	Electrospray ionization
GO	Gene Ontology
GSH	Reduced glutathione
HMGCS1	3-Hydroxy-3-methylglutaryl-CoA synthase 1
IMI	Imidacloprid
KEGG	Kyoto Encyclopedia of Genes and Genomes
MDA	Malondialdehyde
MS/MS	Tandem mass spectrometry
NF-κB	Nuclear factor kappa B
NIH	IMI combined with 100 μg/L PS-NPs
NIL	IMI combined with 25 μg/L PS-NPs
NIM	IMI combined with 50 μg/L PS-NPs
NLRP3	NLR family pyrin domain-containing protein 3
NPS	PS-NPs-only exposure group
PCA	Principal component analysis
PPAR	Peroxisome proliferator-activated receptor
PPI	Protein–protein interaction
PS-NPs	Polystyrene nanoplastics
QC	Quality control
ROS	Reactive oxygen species
RT-qPCR	Reverse-transcription quantitative polymerase chain reaction
SOD	Superoxide dismutase
TNF-α	Tumor necrosis factor alpha
UHPLC	Ultra-high-performance liquid chromatography
VIP	Variable importance in projection
