# Supplementary material for: Transcriptomic and Metabolomic Analyses Reveal Intestinal Lipid Metabolic Disturbance in Loaches Co-Exposed to Polystyrene Nanoplastics and Imidacloprid
Source: Biology (Basel). 2026 Sep 11;15(18):1608. doi: 10.3390/biology15181608 (PMC13604689; doi:10.3390/biology15181608)
Supplement: Supplementary file 1 [file biology-15-01608-s001.zip › Supplementary Table S2.pdf]

Supplementary Table S2. Amplification efficiency and linearity of qPCR assays used in this study.

Gene	Tissue	Slope	Efficiency (%)	R^2
il1b	Liver	-3.3330	99.54	0.9992
il6	Liver	-3.4010	96.80	0.9978
il8	Liver	-3.2870	101.48	0.9926
tnfα	Liver	-3.3650	98.23	0.9869
il4	Liver	-3.4480	94.99	0.9858
Il10	Liver	-3.3180	100.16	0.9981
hmgcs1	Intestine	-3.2960	101.09	0.9890
cel	Intestine	-3.3860	97.39	0.9971
cyp8b1	Intestine	-3.4210	96.03	0.9763
apob	Intestine	-3.3100	100.50	0.9988
apoc1	Intestine	-3.3720	97.95	0.9875
gapdh	Reference	-3.3520	98.76	0.9921

Amplification efficiency was calculated as $E (\%) = [10^{(-1/slope)} - 1] \times 100$ based on serially diluted pooled cDNA standard curves.
