# Supplementary material for: Transcriptomic and Metabolomic Analyses Reveal Intestinal Lipid Metabolic Disturbance in Loaches Co-Exposed to Polystyrene Nanoplastics and Imidacloprid
Source: Biology (Basel). 2026 Sep 11;15(18):1608. doi: 10.3390/biology15181608 (PMC13604689; doi:10.3390/biology15181608)
Supplement: Supplementary file 1 [file biology-15-01608-s001.zip › Supplementary_Table_S1.pdf]

Supplementary Table S1. Statistical analysis of survival and hepatic oxidative-stress biomarkers after 21 days of exposure

Table S1A. Tank-level descriptive statistics, assumption checks, and overall treatment effects

Endpoint	CON	NPS	IMI	NIL	NIM	NIH	Overall test	Exact P	Shapiro-Wilk P	Levene P
CAT (U/g)	36.89 ± 0.36	16.88 ± 1.09	30.29 ± 1.17	19.25 ± 0.22	6.28 ± 0.33	3.94 ± 0.51	F(5,12) = 979.500	3.117×10^{-15}	0.3403	0.2028
SOD (U/g)	89.19 ± 1.13	71.72 ± 0.86	80.39 ± 1.46	79.64 ± 0.48	64.60 ± 0.10	60.94 ± 1.73	F(5,12) = 276.440	5.896×10^{-12}	0.5794	0.0590
MDA (nmol/g)	4.56 ± 0.20	7.17 ± 0.11	5.81 ± 0.44	7.23 ± 0.39	8.47 ± 0.38	13.04 ± 0.08	F(5,12) = 283.651	5.060×10^{-12}	0.4680	0.0634
GSH (μg/g)	75.50 ± 2.76	69.80 ± 0.37	69.28 ± 0.68	57.73 ± 0.47	49.44 ± 2.74	40.89 ± 0.29	Welch F(5,5.405) = 1768.959	1.211×10^{-8}	0.0274	0.0070

Note: Data are presented as mean ± SD of replicate-tank means (n = 3 tanks per treatment). Individual measurements within the same tank were averaged before statistical analysis. CAT, SOD, and MDA were analyzed using one-way ANOVA followed by Tukey–Kramer multiple-comparison tests. Because the GSH data did not satisfy the assumptions of normality and homogeneity of variance, GSH was analyzed using Welch's ANOVA followed by Games–Howell multiple comparisons.

Table S1B. Pairwise comparisons of hepatic oxidative-stress biomarkers

Mean difference is calculated as second group − first group. CAT, SOD, and MDA use Tukey–Kramer-adjusted P values; GSH uses Games–Howell P values.

CAT

Comparison	Mean difference (U/g)	95% CI	Adjusted P
CON vs NPS	−20.01	−21.98 to −18.04	3.08×10^{-12}
CON vs IMI	−6.60	−8.57 to −4.63	1.16×10^{-6}
CON vs NIL	−17.63	−19.60 to −15.67	1.38×10^{-11}
CON vs NIM	−30.61	−32.58 to −28.64	1.81×10^{-14}
CON vs NIH	−32.95	−34.92 to −30.98	6.77×10^{-15}
NPS vs IMI	13.41	11.44 to 15.38	3.49×10^{-10}
NPS vs NIL	2.37	0.41 to 4.34	0.0155
NPS vs NIM	−10.60	−12.57 to −8.63	5.42×10^{-9}
NPS vs NIH	−12.94	−14.91 to −10.97	5.30×10^{-10}
IMI vs NIL	−11.04	−13.00 to −9.07	3.40×10^{-9}
IMI vs NIM	−24.01	−25.98 to −22.04	3.51×10^{-13}
IMI vs NIH	−26.35	−28.32 to −24.38	1.15×10^{-13}
NIL vs NIM	−12.97	−14.94 to −11.01	5.14×10^{-10}
NIL vs NIH	−15.32	−17.28 to −13.35	7.31×10^{-11}
NIM vs NIH	−2.34	−4.31 to −0.37	0.0171

SOD

Comparison	Mean difference (U/g)	95% CI	Adjusted P
CON vs NPS	−17.47	−20.51 to −14.43	2.56×10^{-9}

Comparison	Mean difference (U/g)	95% CI	Adjusted P
CON vs IMI	−8.80	−11.84 to −5.75	5.74×10^{-6}
CON vs NIL	−9.55	−12.59 to −6.51	2.37×10^{-6}
CON vs NIM	−24.59	−27.63 to −21.55	4.66×10^{-11}
CON vs NIH	−28.25	−31.29 to −25.21	9.00×10^{-12}
NPS vs IMI	8.68	5.64 to 11.72	6.63×10^{-6}
NPS vs NIL	7.92	4.88 to 10.96	1.73×10^{-5}
NPS vs NIM	−7.11	−10.16 to −4.07	5.18×10^{-5}
NPS vs NIH	−10.78	−13.82 to −7.74	6.28×10^{-7}
IMI vs NIL	−0.76	−3.80 to 2.29	0.9549
IMI vs NIM	−15.79	−18.83 to −12.75	8.27×10^{-9}
IMI vs NIH	−19.46	−22.50 to −16.42	7.30×10^{-10}
NIL vs NIM	−15.04	−18.08 to −11.99	1.46×10^{-8}
NIL vs NIH	−18.70	−21.74 to −15.66	1.16×10^{-9}
NIM vs NIH	−3.66	−6.71 to −0.62	0.0156

MDA

Comparison	Mean difference (nmol/g)	95% CI	Adjusted P
CON vs NPS	2.61	1.78 to 3.43	2.29×10^{-6}
CON vs IMI	1.25	0.42 to 2.07	0.0029
CON vs NIL	2.67	1.84 to 3.49	1.79×10^{-6}
CON vs NIM	3.91	3.08 to 4.74	2.44×10^{-8}
CON vs NIH	8.47	7.65 to 9.30	2.81×10^{-12}
NPS vs IMI	−1.36	−2.19 to −0.53	0.0014
NPS vs NIL	0.06	−0.77 to 0.89	0.9998
NPS vs NIM	1.30	0.48 to 2.13	0.0020
NPS vs NIH	5.87	5.04 to 6.70	2.17×10^{-10}
IMI vs NIL	1.42	0.59 to 2.25	0.0010
IMI vs NIM	2.66	1.84 to 3.49	1.81×10^{-6}
IMI vs NIH	7.23	6.40 to 8.05	1.86×10^{-11}
NIL vs NIM	1.24	0.42 to 2.07	0.0030
NIL vs NIH	5.81	4.98 to 6.64	2.45×10^{-10}
NIM vs NIH	4.57	3.74 to 5.39	4.08×10^{-9}

GSH

Comparison	Mean difference (μg/g)	95% CI	Games-Howell P
CON vs NPS	−5.70	−18.53 to 7.13	0.2289
CON vs IMI	−6.22	−18.27 to 5.83	0.1898
CON vs NIL	−17.77	−30.39 to −5.15	0.0248
CON vs NIM	−26.06	−36.71 to −15.41	0.0019
CON vs NIH	−34.62	−47.60 to −21.64	0.0070
NPS vs IMI	−0.52	−2.99 to 1.96	0.8340
NPS vs NIL	−12.07	−13.75 to −10.39	3.72×10^{-5}
NPS vs NIM	−20.36	−33.05 to −7.67	0.0193
NPS vs NIH	−28.92	−30.25 to −27.58	5.85×10^{-7}
IMI vs NIL	−11.55	−13.96 to −9.14	2.49×10^{-4}
IMI vs NIM	−19.84	−31.75 to −7.93	0.0165
IMI vs NIH	−28.40	−31.01 to −25.79	8.27×10^{-5}
NIL vs NIM	−8.29	−20.77 to 4.19	0.1123
NIL vs NIH	−16.85	−18.52 to −15.18	2.55×10^{-5}
NIM vs NIH	−8.56	−21.40 to 4.29	0.1093

Table S1C. Survival analysis

Group	n	Final survival (%)	95% CI
CON	90	100.0	95.9–100.0
NPS	90	95.2	88.6–98.1
IMI	90	95.2	88.6–98.1
NIL	90	90.5	82.7–95.0
NIM	90	90.5	82.7–95.0
NIH	90	85.7	77.0–91.5

Overall Kaplan–Meier log-rank test: $\chi^2 = 18.19$, df = 5, P = 0.0027.

Note: Survival analysis included 90 fish per treatment. Confidence intervals for final survival proportions are 95% confidence intervals.
