# Supplementary material for: Transcriptomic and Metabolomic Analyses Reveal Intestinal Lipid Metabolic Disturbance in Loaches Co-Exposed to Polystyrene Nanoplastics and Imidacloprid
Source: Biology (Basel). 2026 Sep 11;15(18):1608. doi: 10.3390/biology15181608 (PMC13604689; doi:10.3390/biology15181608)
Supplement: Supplementary file 1 [file biology-15-01608-s001.zip › Supplementary_Table_S3.pdf]

Supplementary Table S3. Fish-level semi-quantitative histopathological scores (n = 4 fish per treatment).

Gill: fish-level lesion scores

Group	Fish 1	Fish 2	Fish 3	Fish 4	n	Mean ± SD	Median	Range
CON	0	0	0	0	4	0.00 ± 0.00	0.0	0–0
NPS	1	1	1	1	4	1.00 ± 0.00	1.0	1–1
IMI	1	2	1	2	4	1.50 ± 0.58	1.5	1–2
NIL	1	1	1	1	4	1.00 ± 0.00	1.0	1–1
NIM	2	1	2	2	4	1.75 ± 0.50	2.0	1–2
NIH	3	3	3	3	4	3.00 ± 0.00	3.0	3–3

Intestine: fish-level lesion scores

Group	Fish 1	Fish 2	Fish 3	Fish 4	n	Mean ± SD	Median	Range
CON	0	0	0	0	4	0.00 ± 0.00	0.0	0–0
NPS	1	1	1	1	4	1.00 ± 0.00	1.0	1–1
IMI	2	2	2	1	4	1.75 ± 0.50	2.0	1–2
NIL	2	2	2	1	4	1.75 ± 0.50	2.0	1–2
NIM	2	2	1	1	4	1.50 ± 0.58	1.5	1–2
NIH	3	3	3	2	4	2.75 ± 0.50	3.0	2–3

Liver: fish-level lesion scores

Group	Fish 1	Fish 2	Fish 3	Fish 4	n	Mean ± SD	Median	Range
CON	0	0	0	0	4	0.00 ± 0.00	0.0	0–0
NPS	1	1	1	1	4	1.00 ± 0.00	1.0	1–1
IMI	1	1	1	1	4	1.00 ± 0.00	1.0	1–1
NIL	1	1	1	1	4	1.00 ± 0.00	1.0	1–1
NIM	2	1	2	1	4	1.50 ± 0.58	1.5	1–2
NIH	3	3	3	2	4	2.75 ± 0.50	3.0	2–3

Note: Each Fish 1 – 4 value represents the mean lesion score obtained from three to five non-overlapping 20× microscopic fields from one individual fish.Scoring scale: 0 = no obvious lesion; 1 = mild focal change; 2 = moderate lesion; 3 = severe/extensive architectural disruption or inflammatory change.
